# A Comprehensive Approach to Sample Preparation for Electron Microscopy and the Assessment of Mitochondrial Morphology in Tissue and Cultured Cells

**DOI:** 10.1002/adbi.202200202

**Published:** 2023-05-04

**Authors:** Antentor Hinton, Prasanna Katti, Trace A. Christensen, Margaret Mungai, Jianqiang Shao, Liang Zhang, Sergey Trushin, Ahmad Alghanem, Adam Jaspersen, Rachel E. Geroux, Kit Neikirk, Michelle Biete, Edgar Garza Lopez, Bryanna Shao, Zer Vue, Larry Vang, Heather K. Beasley, Andrea G. Marshall, Dominique Stephens, Steven Damo, Jessica Ponce, Christopher K. E. Bleck, Innes Hicsasmaz, Sandra A. Murray, Ranthony A. C. Edmonds, Andres Dajles, Young Do Koo, Serif Bacevac, Jeffrey L. Salisbury, Renata O. Pereira, Brian Glancy, Eugenia Trushina, E. Dale Abel

**Affiliations:** Department of Molecular Physiology and Biophysics, Vanderbilt University, 2201 West End Ave, Nashville, TN 37235, USA; Microscopy and Cell Analysis Core Facility, Mayo Clinic, 200 First Street SW, Rochester, MN 55905, USA; Department of Internal Medicine, University of Iowa – Carver College of Medicine, 200 Hawkins Drive, Iowa City, IA 52242, USA; Fraternal Order of Eagles Diabetes Research Center, 169 Newton Rd, Iowa City, IA 52242, USA; National Heart, Lung, and Blood Institute, National Institutes of Health, 9000 Rockville Pike, Bethesda, MD 20892, USA; Microscopy and Cell Analysis Core Facility, Mayo Clinic, 200 First Street SW, Rochester, MN 55905, USA; Department of Internal Medicine, University of Iowa – Carver College of Medicine, 200 Hawkins Drive, Iowa City, IA 52242, USA; Fraternal Order of Eagles Diabetes Research Center, 169 Newton Rd, Iowa City, IA 52242, USA; Central Microscopy Research Facility, University of Iowa, Iowa City, IA 52242, USA; Department of Neurology, Mayo Clinic, 200 First Street SW, Rochester, MN 55905, USA; Department of Neurology, Mayo Clinic, 200 First Street SW, Rochester, MN 55905, USA; Eastern Region, King Abdullah International Medical Research Center, King Saud bin Abdulaziz University for Health Sciences, Riyadh 11481 Al Hasa, Saudi Arabia; Department of Internal Medicine, Division of Cardiology, Washington University in St. Louis, 1 Brookings Dr, St. Louis, MO 63130, USA; Microscopy and Cell Analysis Core Facility, Mayo Clinic, 200 First Street SW, Rochester, MN 55905, USA; Department of Neurology, Mayo Clinic, 200 First Street SW, Rochester, MN 55905, USA; College of Natural and Health Sciences, University of Hawaii at Hilo, 200 West Kawili St, Hilo, HI 96720, USA; College of Natural and Health Sciences, University of Hawaii at Hilo, 200 West Kawili St, Hilo, HI 96720, USA; Department of Internal Medicine, University of Iowa – Carver College of Medicine, 200 Hawkins Drive, Iowa City, IA 52242, USA; Department of Molecular Physiology and Biophysics, Vanderbilt University, 2201 West End Ave, Nashville, TN 37235, USA; Department of Molecular Physiology and Biophysics, Vanderbilt University, 2201 West End Ave, Nashville, TN 37235, USA; Department of Molecular Physiology and Biophysics, Vanderbilt University, 2201 West End Ave, Nashville, TN 37235, USA; Department of Biochemistry, Cancer Biology, Neuroscience and Pharmacology, School of Graduate Studies and Research, Meharry Medical College, Nashville, TN 37208, USA; Department of Molecular Physiology and Biophysics, Vanderbilt University, 2201 West End Ave, Nashville, TN 37235, USA; Department of Molecular Physiology and Biophysics, Vanderbilt University, 2201 West End Ave, Nashville, TN 37235, USA; Department of Life and Physical Sciences, Fisk University, Nashville, TN 37208, USA; Department of Molecular Physiology and Biophysics, Vanderbilt University, 2201 West End Ave, Nashville, TN 37235, USA; Department of Life and Physical Sciences, Fisk University, Nashville, TN 37208, USA; School of Medicine, University of Utah, 30 N 1900 E, Salt Lake City, UT 84132, USA; National Heart, Lung, and Blood Institute, National Institutes of Health, 9000 Rockville Pike, Bethesda, MD 20892, USA; Department of Internal Medicine, University of Iowa – Carver College of Medicine, 200 Hawkins Drive, Iowa City, IA 52242, USA; Fraternal Order of Eagles Diabetes Research Center, 169 Newton Rd, Iowa City, IA 52242, USA; Department of Cell Biology, University of Pittsburgh, Pittsburgh, PA 15206, USA; Department of Mathematics, Ohio State University, 281 W Lane Ave, Columbus, OH 43210, USA; Department of Internal Medicine, University of Iowa – Carver College of Medicine, 200 Hawkins Drive, Iowa City, IA 52242, USA; Department of Internal Medicine, University of Iowa – Carver College of Medicine, 200 Hawkins Drive, Iowa City, IA 52242, USA; Fraternal Order of Eagles Diabetes Research Center, 169 Newton Rd, Iowa City, IA 52242, USA; Department of Internal Medicine, University of Iowa – Carver College of Medicine, 200 Hawkins Drive, Iowa City, IA 52242, USA; Fraternal Order of Eagles Diabetes Research Center, 169 Newton Rd, Iowa City, IA 52242, USA; Department of Biochemistry and Molecular Biology, Mayo Clinic, 200 First Street SW, Rochester, MN 55905, USA; Microscopy and Cell Analysis Core Facility, Mayo Clinic, 200 First Street SW, Rochester, MN 55905, USA; Fraternal Order of Eagles Diabetes Research Center, 169 Newton Rd, Iowa City, IA 52242, USA; Department of Internal Medicine, University of Iowa – Carver College of Medicine, 200 Hawkins Drive, Iowa City, IA 52242, USA; National Institute of Arthritis and Musculoskeletal and Skin Diseases, National Institutes of Health, 9000 Rockville Pike, Bethesda, MD 20892, USA; National Heart, Lung, and Blood Institute, National Institutes of Health, 9000 Rockville Pike, Bethesda, MD 20892, USA; Department of Molecular Pharmacology and Experimental Therapeutics, Mayo Clinic, 200 First Street SW, Rochester, MN 55905, USA; Department of Neurology, Mayo Clinic, 200 First Street SW, Rochester, MN 55905, USA; Fraternal Order of Eagles Diabetes Research Center, 169 Newton Rd, Iowa City, IA 52242, USA; Department of Internal Medicine, University of Iowa – Carver College of Medicine, 200 Hawkins Drive, Iowa City, IA 52242, USA; Department of Medicine, UCLA, 757 Westwood Plaza, Suite 7236, David Geffen School of Medicine, Los Angeles, CA 90095, USA

**Keywords:** automated serial block-face SEM, focused ion beam SEM, mitochondria-endoplasmic reticulum communication, mitochondrial dynamics, mitochondrial morphology, serial-section TEM

## Abstract

Mitochondria respond to metabolic demands of the cell and to incremental damage, in part, through dynamic structural changes that include fission (fragmentation), fusion (merging of distinct mitochondria), autophagic degradation (mitophagy), and biogenic interactions with the endoplasmic reticulum (ER). High resolution study of mitochondrial structural and functional relationships requires rapid preservation of specimens to reduce technical artifacts coupled with quantitative assessment of mitochondrial architecture. A practical approach for assessing mitochondrial fine structure using two dimensional and three dimensional high-resolution electron microscopy is presented, and a systematic approach to measure mitochondrial architecture, including volume, length, hyperbranching, cristae morphology, and the number and extent of interaction with the ER is described. These methods are used to assess mitochondrial architecture in cells and tissue with high energy demand, including skeletal muscle cells, mouse brain tissue, and *Drosophila* muscles. The accuracy of assessment is validated in cells and tissue with deletion of genes involved in mitochondrial dynamics.

## 1. Introduction

Mitochondria are responsible for meeting the energetic and metabolic demands required for cellular functions.^[1]^ Determination of mitochondrial size, shape, network organization, and interactions with other organelles could contribute toward the understanding of normal and disease mechanisms and monitoring of therapeutic efficacy.^[2–12]^ Mitochondrial number and shape are determined in part by fusion and fission cycles.^[2–7]^ Fusion is mediated by the fusion proteins optic atrophy 1 (OPA1) and mitofusin-1 and −2 (MFN1 and MFN2, respectively).^[8,13,14]^ Likewise, fission machinery includes dynamin-related protein 1 (DRP-1) and its receptors mitochondrial fission 1 protein and mitochondrial fission factor, and the mitochondrial dynamics proteins MiD49 and MiD51.^[5,7,8,10,14–16]^ Mitochondria interact with other intracellular organelles, including the endoplasmic reticulum (ER), through direct mitochondria–ER contacts (MERCs).^[1,17–23]^ Mitochondria also interact with lipid droplets (LDs) through either transient, kiss-and-run contacts, or the longer-lived LD-anchored mitochondria contacts.^[2]^ Mitochondria further communicate with the nucleus via the release of metabolic intermediates that mediate transcriptional regulation.^[1,4,17–24]^ Furthermore, mitochondria form dynamic branching networks and connections via nanotunnels or mitochondria-on-a-string (MOAS).^[25–28]^ Nanotunnels and MOAS link mitochondrial elements together in response to energetic stress, hypoxia, and other physiological and environmental changes. Nanotunnels and MOAS are often detected in disease states, such as Alzheimer’s disease (AD).^[25,29,30]^ Their formation is thought to represent a mechanism for promoting mitochondrial communication and protecting mitochondria against fragmentation and lysosomal degradation.^[25–27,31,32]^ These unique structures are often formed within minutes of exposure to hypoxic conditions, therefore specimen preparation methods for electron microscopy should minimize the potential for exposure of cells and tissue to hypoxic stress. Specifically, development of methodologies capable of reducing hypoxic artifacts during specimen preparation and the development of standardized approaches for quantifying changes in a consistent manner are imperative for accurate assessment of mitochondrial structure, and to promote the reproducible evaluation of samples for comparison across laboratories.

Since early studies of mitochondria using electron microscopy (EM),^[33,34]^ a myriad of methods have been developed for specimen preparation to best preserve the native organellar morphology.^[24,35–42]^ However, no clear consensus has been reached regarding which preparation method most reliably ensures reproducible and artifact-free resolution of mitochondrial morphology in cells or tissue. Early studies of mitochondrial structure were limited to two dimensional (2D) optical or EM imaging techniques. More recently, specialized instrumentation has been employed to generate high-resolution volume renderings using three dimensional (3D) EM. The novel MOAS phenotype was only recently discovered using 3D EM reconstruction and is not obvious when using conventional 2D transmission EM (TEM).^[25]^ This report details a systematic approach to the characterization of 3D mitochondrial morphology, including the identification of MOAS in multiple tissue types.

The objectives of this study were (1) to develop an optimized approach for specimen handling and fixation that preserves mitochondrial morphology in cells and tissue for EM evaluation; (2) to detail comprehensive methodology for quantifying organellar morphological characteristics using TEM micrographs and 3D EM reconstructions; and (3) to validate the reproducibility of these methods in describing morphological changes that occur in subcellular organelles. Specifically, we focused on quantifying mitochondrial morphological indices,^[2–7]^ such as mitochondrial volume, cristae morphology,^[8–12]^ and the length and percentage coverage of MERCs.^[1,17,19,20,43]^ We optimized specimen preparation conditions to reduce technical artifacts, and validated these methods in cultured cells, mouse brain tissue, and *Drosophila*. Multiple models utilized in the study, including murine skeletal muscle myotubes, murine cardiomyocytes, murine brain tissue, and *Drosophila* indirect flight muscles, allowed validation of the protocols in cells and tissue with highly active mitochondria. We conducted gene deletion for known mitochondrial dynamics proteins, including OPA1, MFN2, and DRP-1, to further demonstrate that developed protocols provide reliable and reproducible monitoringofchangesinmitochondrialdynamics.3DEMreconstruction was applied to manual serial-section TEM, automated serial block-face (SBF)-scanning EM (SEM), or focused ion beam (FIB)-SEM. Tools, including widely available open-source image analysis platforms ImageJ (FIJI) and Reconstruct, were used to quantify EM micrographs and display 3D images following segmentation, respectively.^[25,44,45]^ Additional software included Ilastik^[1]^ and Amira,^[46]^ which were used for the reconstruction of FIB-SEM and SBF-SEM acquisitions, respectively. We present a comprehensive approach to artifact-free sample preparation and analyses of mitochondrial morphology in multiple cells and tissue with diverse mitochondrial structures for 3D EM reconstructions.

## 2. Results

### 2.1. Preparation of Cultured Cells for Ultrastructural Studies Using EM

**Figure 1.**
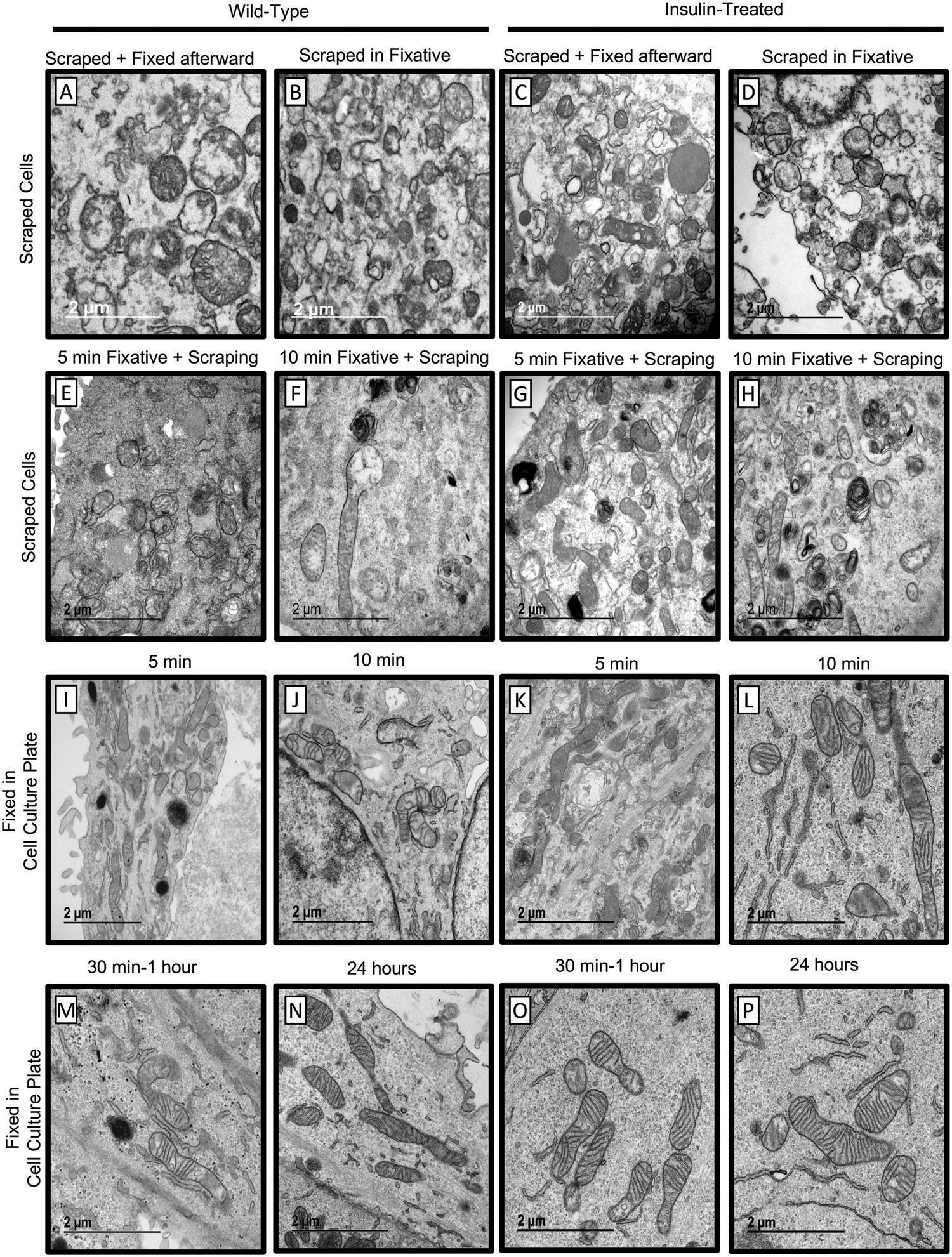
The effect of different methods for fixing cultured cells on mitochondrial morphology determined by TEM. A–D) Live cells were scraped directly into McDowell Trump’s fixative. E,G) Cells were fixed for 5 min and then scraped in McDowell Trump’s fixative. F,H) Cells were fixed for 10 min and then scraped in McDowell Trump’s fixative. I,K) Cells were fixed for 5 min in McDowell Trump’s fixative without scraping. J,L) Cells were fixed for 10 min in McDowell Trump’s fixative without scraping. M,O) Cells were fixed for 30–60 min in McDowell Trump’s fixative without scraping. N,P) Cells were fixed for 24 h in McDowell Trump’s fixative without scraping. Cells were treated with insulin for 2 h before fixation.

We first established the optimal conditions for preserving mitochondrial ultrastructure in cultured cells for TEM examination that allow estimating fine details of mitochondrial and cristae morphology. Insulin stimulation can increase mitochondrial fusion in cultured cardiomyocytes and myotubes, resulting in larger, fused mitochondria with increased cristae density.^[5]^ Myotubes prepared from differentiated skeletal muscle satellite cells were stimulated with insulin for 2 h prior to fixation. This regimen increased mitochondrial size and cristae density and was used for the validation of specimen preparation. Eight different fixation methods testing various cell harvesting and fixation techniques were compared to determine the best approach to preserve mitochondrial and cristae morphology (Figure 1A–P).

### 2.2 . Effect of Cell Scraping on Fixation

Skeletal muscle myotubes were grown in Matrigel-coated 6well plates (Figure 1). Cells were treated with insulin (2 h at 10 nmol L^−1^)^[2]^ or vehicle. We first determined whether scraping of live cells directly into the fixative preserves mitochondrial morphology. All experiments were conducted at room temperature. Live cells were either scraped immediately into the McDowell Trump’s fixative (Figure 1A–D) or pre-fixed for 5 min (Figure 1E,G) or 10 min (Figure 1F,H) before scraping into the fixative. Each of these methods resulted in discernable structural artifacts. Scraping cells before fixation altered the plasma membrane and damaged mitochondria, resulting in the appearance of discontinuities in membranes (Figure 1A–D). Short fixation time before scraping also resulted in incomplete fixation where all samples contained mitochondria with disrupted cristae integrity, indicating insufficient preservation for TEM analysis (Figure 1A–H). These observations suggest that scraping during cell harvesting damages the plasma membrane and disrupts mitochondrial integrity and therefore should be avoided.

We next determined whether fixation on the tissue culture plate without scraping improves mitochondrial preservation. Skeletal muscle myotubes were fixed with warm McDowell Trump’s fixative added directly to the plate for 5 min (Figure 1I,K), 10 min (Figure 1J,L), 30–60 min (Figure 1M,O), or 24 h (Figure 1N,P). Consistent with the previous findings (Figure 1E,G), mitochondria were poorly preserved after 5 or 10 min of fixation (Figure 1I–L). In contrast, fixation for 30–60 min or 24h(Figure1M–P) greatly improved mitochondrial preservation. These experiments indicate that fixing cells directly on a plate for at least 30 min without scraping was the most effective method to preserve mitochondria for EM examination and should be considered for cultured cells.

### 2.3. Systematic Quantification of Mitochondrial Morphology and Interactions with Other Organelles

**Figure 2.**
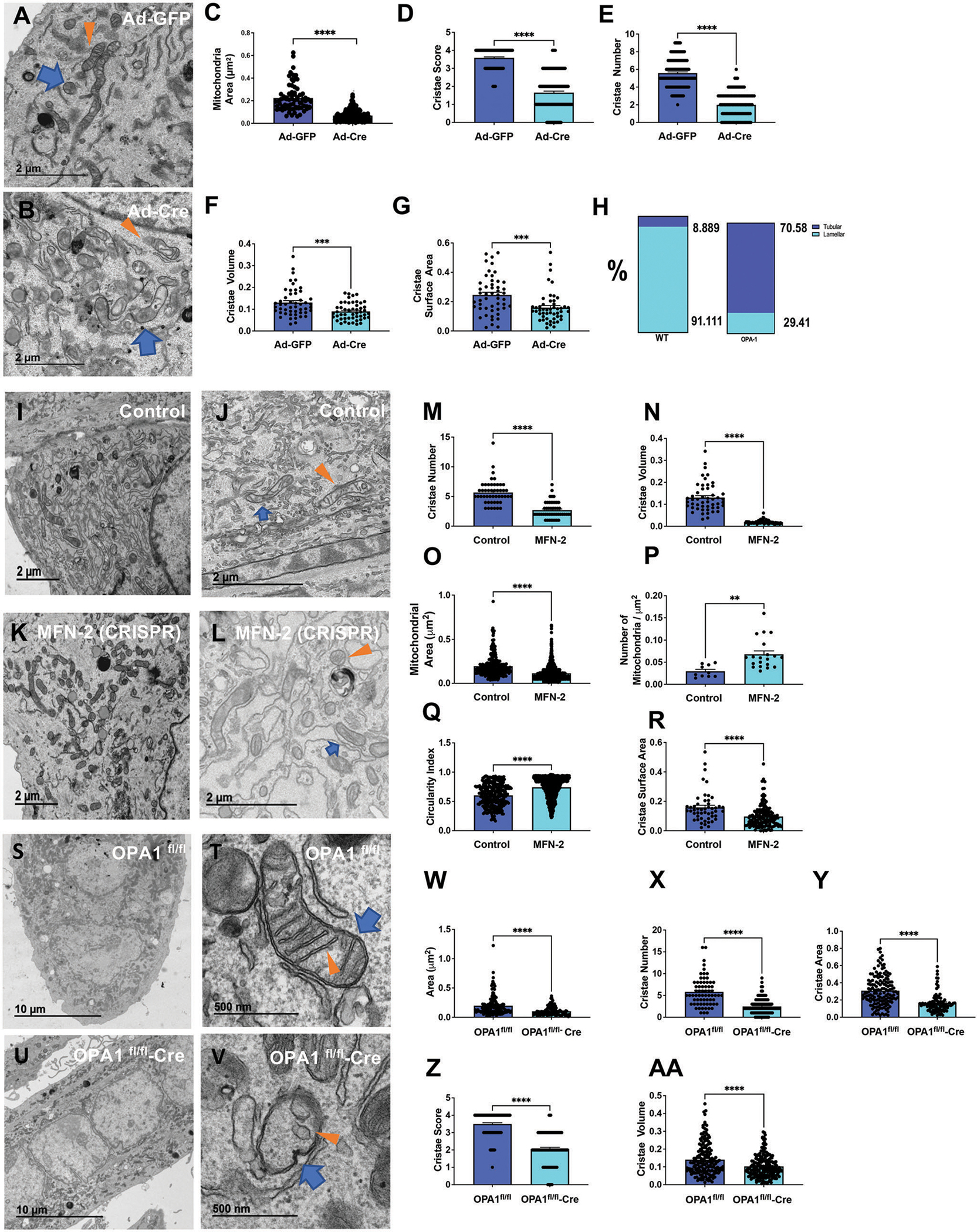
Quantification of mitochondrial characteristics in *Opa1*-deficient murine skeletal muscle myoblasts and *Mfn2*-deficient or *Opa1*-deficient murine skeletal muscle myotubes. Ultrastructure of A) control and B) *Opa1*-deficient primary skeletal muscle myoblasts. Quantification of C) mitochondrial area, D) cristae score, E) cristae number, F) cristae volume, and G) cristae surface area in control and *Opa1*-deficient primary skeletal muscle myoblasts. H) Percentage of tubular and lamellar cristae in control and *Opa1*-deficient primary skeletal muscle myoblasts. Ultrastructure of I,J) control and K,L) *Mfn2*-deficient primary skeletal muscle myotubes. Quantification of M) cristae number, N) cristae volume, O) mitochondrial area, P) number of mitochondria per μm^2^, Q) circularity index, and R) cristae surface area in control and *Mfn2*-deficient skeletal muscle myotubes. Ultrastructure of S,T) control and U,V) *Opa1*-deficient primary skeletal muscle myotubes. W) Quantification of area, X) cristae number, Y) cristae area, Z) cristae score, and AA) cristae volume in control and *Opa1*-deficient skeletal muscle myotubes. Scale bars: A,B,I–L) 2 μm; S,U) 10 μm; T,V) 500 nm. Recombinant eGFP adenovirus (Ad-GFP) serves as a control while adenovirus-cre represents the experimental knockout. *Opa1*^*fl/fl*^ represents a control while *Opa1*^*fl/fl*^-*cre* represents floxed deletion of *Opa1*. Blue arrows indicate mitochondria; orange arrows indicate cristae. Significance was determined using Welch’s *t*-test. **p < 0.01, ***p < 0.001, ****p < 0.0001.

**Figure 3.**
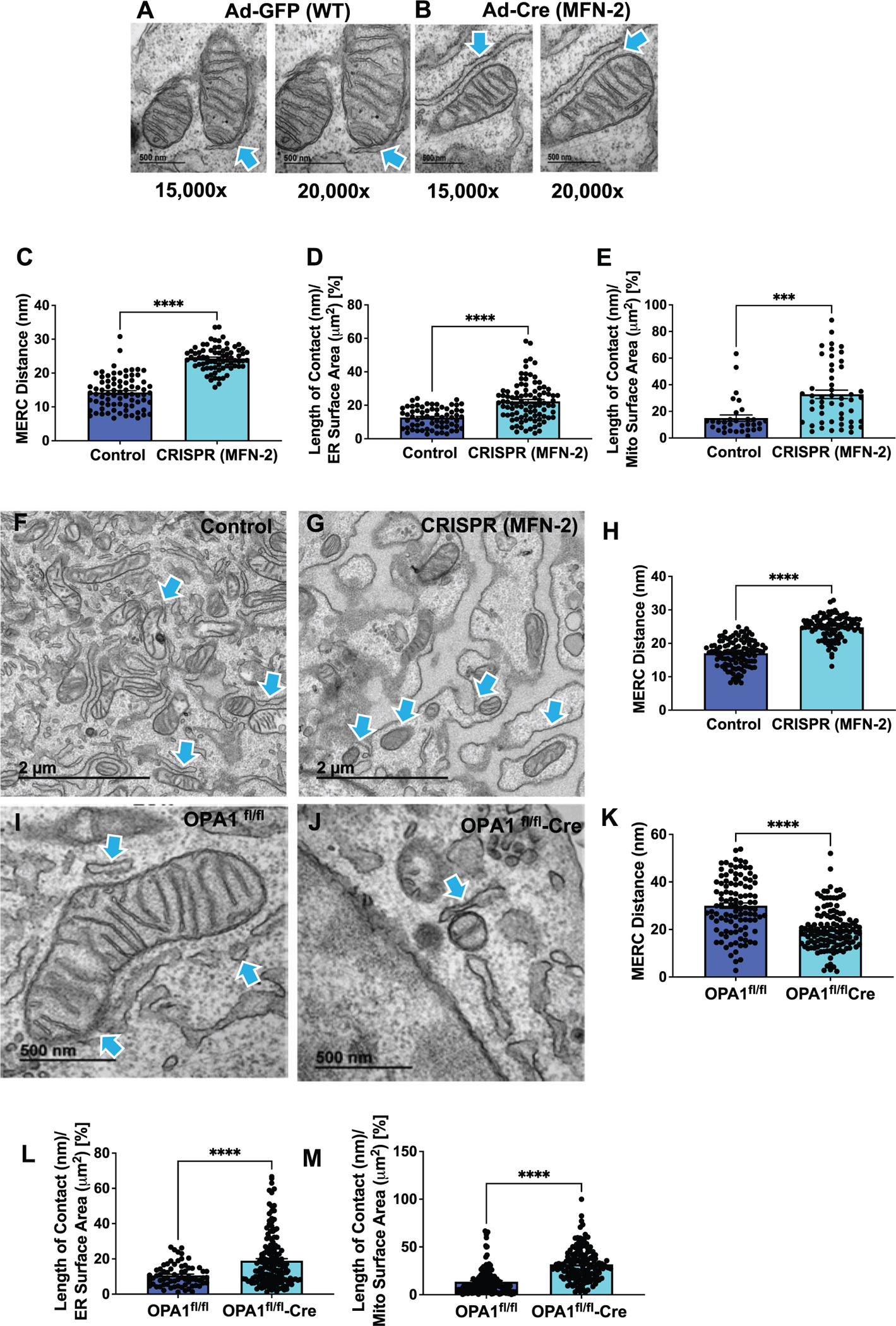
MERCs ultrastructure in *Mfn2*-deficient mouse fibroblasts and *Opa1*-deficient primary skeletal muscle myotubes. A,B) Control and *Mfn2*-deficient primary fibroblasts were imaged using TEM at 15 000× and 20 000× magnification to quantify MERCs. Blue arrows indicate MERCs. Quantification of C) MERCs distance, D) len) p E) len expin control CRISPR and *Mfn2*-deficient primary fibroblasts. Ultrastructure of F) control and G) CRISPR-generated *Mfn2*-deficient primary myotubes. Blue arrows indicate MERCs. H) MERCs distance in control CRISPR and *Mfn2*-deficient primary myotubes. Ultrastructure of I) control and J) *Opa1*-deficient primary skeletal muscle myotubes. Quantification of K) MERCs distance, L) length of contact (nm) per ER surface area (μm^2^) percentage, and M) length of contact (nm) per mit surface area (μm^2^) expressed as a percentage in control and *Opa1*-deficient primary skeletal muscle myotubes. Scale bars: A,B) 500 nm; F,G) 2 μm; I,J) 500 nm. Recombinant eGFP adenovirus (Ad-GFP) serves as a control while adenovirus-cre represents the experimental knockout. *Opa1*^*fl/fl*^ represents a control, while *Opa1*^*fl/fl*^-*cre* represents floxed deletion of *Opa1*. Significance was determined using a Mann–Whitney -test. ***p < 0.001, ****p < 0.0001.

We next applied the established fixation method to describe changes in mitochondrial morphology and the interaction with other organelles in response to hormonal and genetic manipulations (Figures2, 3, S1, and S2). Details describing how mitochondrial morphology was quantified in TEM images are reported in the Experimental Section.

To determine whether fixation method allows the accurate detection of morphological changes, we used murine skeletal muscle myoblasts and myotubes with genetic ablation of *Opa1, Mfn2*, and *Drp1*. OPA1 is a mitochondrial GTPase that maintains cristae morphology and is responsible for inner mitochondrial membrane fusion.^[14,47]^ We first quantified changes in mitochondrial morphology and cristae integrity in mouse primary myoblasts with *Opa1* knock-down.^[5,44,46]^ Cells were fixed for 1 h without scraping. Reduction in OPA1 expression resulted in a decrease in mitochondrial area (Figure 2A–C), reduced cristae score, cristae number, volume, and cristae surface area compared to control cells expressing wild-type (WT) levels of OPA1 (Figure 2D–G). Compared to control cells, *Opa1*-deficient myoblasts demonstrated an increase in tubular cristae and a decrease in lamellar cristae (Figure 2H). These structural changes are consistent with the expected consequences of *Opa1* knockdown^[8,44,46–50]^ and thereby support protocol validation for comprehensive characterization of mitochondrial morphology.

MFN2 is a mitochondrial outer membrane GTPase responsible for mitochondrial outer membrane fusion.^[17,20]^ Previous studies have shown cristae remodeling in response to the loss of MFN2 expression.^[20,51,52]^ DRP-1 is a fission protein that is activated by cellular stress.^[16]^ DRP-1 also has an important role in calcium uptake, and the loss of DRP-1 expression results in the accumulation of enlarged mitochondria and loss of mitochondrial function.^[7]^ We next verified whether the fixation protocol established here allows detection of changes caused by *Mfn2, Opa1*, and *Drp1* ablation. Transient ablation of one of the fission/fusion genes, *Mfn2, Opa1*, and *Drp1*, in primary mouse myotubes was achieved using the clustered regularly interspaced short palindromic repeat (CRISPR)-Cas9 system. Western blot analysis and quantitative PCR confirmed the efficiency of ablation (Figure S1A–D, Supporting Information). Compared to control cells, *Mfn2*-ablated primary myotubes had reduced cristae number, cristae volume, cristae surface area, and mitochondrial area (Figure 2I–O,R). In contrast, the number of mitochondria and the circularity index increased (Figure 2P,Q). The ablation of *Opa1* in primary myotubes using Cre recombinase (Figure 2S–V) produced alteration of mitochondria and cristae architecture like that observed in *Opa1*-knockdown myoblasts (Figure 2A–H). Loss of *Opa1* resulted in a decrease in the mitochondrial area, cristae number, cristae area, cristae score, and cristae volume (Figure 2W–AA). Conversely, ablation of *Drp1* (Figure S1A,C, Supporting Information) resulted in increased mitochondrial area and length (Figure S1E–L, Supporting Information). Interestingly, while the cristae score and surface area decreased, suggesting fewer well formed and smaller cristae, the number of cristae increased (Figure S1M–O, Supporting Information). These data suggest that the fixation protocol optimized for cells allows accurate detection of structural changes in mitochondria in response to changes in expression of fission/fusion proteins.

### 2.4. Determining the Effect of MFN2, OPA1, and DRP-1 Deficiency on MERCs

MERCs are contacts formed between mitochondria and the ER when the organelles are juxtaposed at a distance that ranges from ≈10 to ≈50 nm.^[11]^ MERCs operate as platforms that regulate mitochondrial dynamics, autophagy, lipid homeostasis, calcium signaling, and other important cellular pathways essential for organismal health.^[11,53]^ MERCs are modulated by multiple tethering partners, including fission and fusion proteins OPA1, MFN2, and DRP-1.^[20,48,54–56]^ The ability to accurately determine changes in MERCs number and architecture could provide insight into mechanisms of multiple human diseases and efficacy of therapeutic interventions.^[57]^

While several studies reported that the ablation of *Mfn2* alters MERCs distance,^[6,11,12]^ others have disputed these findings.^[17,58]^ Since the discrepancy may be due to the suboptimal fixation methods,^[13,15,23,58–60]^ we measured the distance between rough ER and mitochondria in *Mfn2*-deficient myotubes using optimized fixation protocol (Figure 3). We also examined the impact of reduced expression of DRP-1 and OPA1 on MERCs. The architecture of mitochondria-ER contacts varied, ranging from a punctate contact site to extensive connections of the membranes. Therefore, when assessing MERCs, it is important to consider various measurements, including the distance between mitochondria and the ER and the percent of coverage between the two organelles. In quantifying MERCs, we used the definition provided by Giacomello and Pellegrini,^[11]^ which classifies MERCs width within 50 nm.^[11]^ The ablation of *Mfn2* in mouse fibroblasts using Cre recombinase^[61–65]^ (Figure 3A,B) increased MERCs distance compared to WT cells, consistent with the expected lack of tethering due to the loss of one of the important tethering proteins (Figure 3C). We next measured the percent of the total mitochondria or the ER surface that was involved in MERCs. We found an increase in the percentage of coverage for both the mitochondrial and the ER surfaces involved in MERCs in *Mfn2*-knockout (KO)cells relative to control fibroblasts (Figure3D,E).^[6]^ Similar increase in MERCs distance was observed in primary myotubes with CRISPR-Cas9 ablation of *Mfn2* (Figure 3F–H). We also performed MERCs quantification in *Opa1*-deficient primary skeletal muscle myotubes (Figure 3I–K). Contrary to changes observed after the *Mfn2* KO, the deletion of *Opa1* resulted in decreased MERCs distance (Figure 3K). However, similar to the *Mfn2*, *Opa1* deletion resulted in increased percent of MERCs coverage of both the ER and mitochondria relative to control myotubes (Figure 3L,M). We further performed MERCs quantification in *Drp1*-deficient murine myotubes^[66]^ (Figure S2, Supporting Information). Similar to *Mfn2* deletion, there was an increase in MERCs distance and ER surface area (Figure S2E,F, Supporting Information). These observations suggest that MERCs distance do not always correlate with MERCs coverage, indicating a complex and dynamic relationship between mitochondria and the ER.^[14]^ These results demonstrate that optimal fixation retained organellar and sub-organellar features associated with gene knockdown.

### 2.5. Tissue Preparation for Ultrastructural Studies Using EM

**Figure 4.**
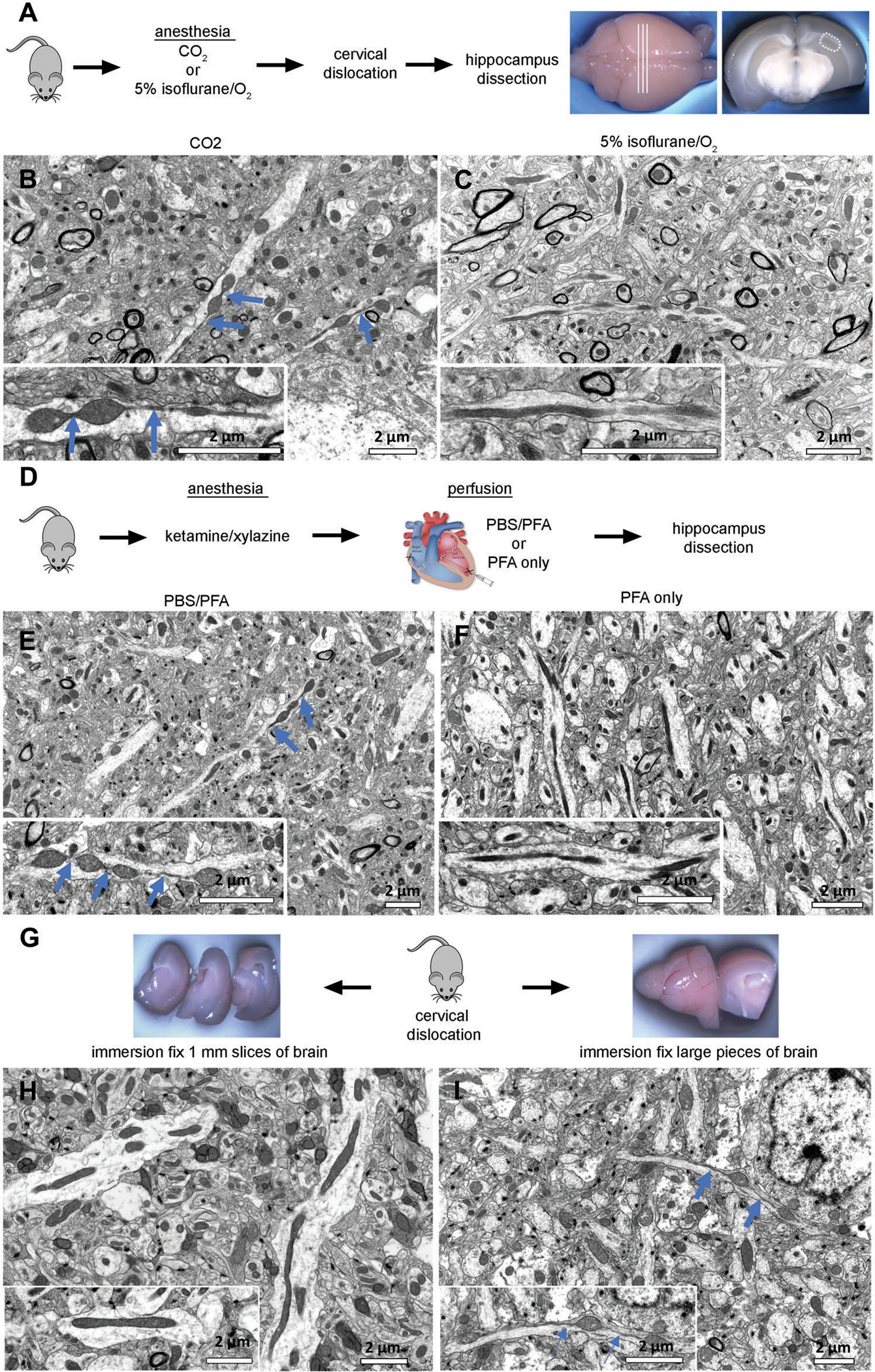
Evaluation of conditions to reduce artifacts in tissue preparation to assay mitochondrial morphology using EM. A) Wild-type (WT) mice were sacrificed by cervical dislocation after anesthesia with CO_2_ or 5% isoflurane/oxygen inhalation. Fresh brains were removed and cut into coronal sections. The CA1 hippocampal region (hipp) from each section was dissected and further processed for TEM or scanning electron microscopy (SEM). B) CO_2_ exposure for 3 min was sufficient to induce MOAS formation in hippocampal tissue of WT mice. Scale bar: 2 μm. C) Mitochondria in hippocampus from mice administered 5% isoflurane prior to cervical dislocation and subsequent dissection maintained a normal shape and size typical for WT mice. Scale bar: 2 μm. D) WT mice were anesthetized using ketamine/xylazine, followed by cardiac perfusion with either phosphate-buffered saline (PBS) an 4% paraformaldehyde (PFA) or 4% PFA only. Mice were euthanized by cervical dislocation, and brain removed and fixed in Trump’s solution overnight. The next day, brains were cut into coronal sections. The CA1 hippocampal region (hipp) from each section was dissected as shown in (A) and subjected to further processing for TEM or SEM. E,F) Cardiac perfusion preceded by PBS flush induced MOAS in hippocampal tissue of WT mice. Perfusion without PBS flush did not cause MOAS. Scale bar: 2 μm. G) WT mice were euthanized by cervical dislocation without prior anesthesia. Brains were removed, and one hemisphere was cut into 1-mm-thick slices (left), while the other hemisphere was cut into two halves (right). Tissues were subjected to immersion fixation in Trump’s solution overnight. H) Tissues cut in thin slices were free of MOAS, I) while larger pieces of tissue displayed pronounced MOAS. Scale bars: 2 μm. Blue arrows indicate MOAS.

To broaden the scope beyond cells, we determined optimal techniques for fixation of mouse tissue. Commonly used techniques for collecting and fixing tissue for EM analysis include multiple methods of anesthesia, followed by cardiac perfusion. During cardiac perfusion, phosphate-buffered saline (PBS) buffer is flushed through the body to remove erythrocytes from blood vessels before perfusing the fixative for a whole-body fixation. Common anesthetics include ketamine/xylazine injections, CO_2_ inhalation, or inhalation of a 5% isoflurane/oxygen mixture. The advantage of the cardiac perfusion with fixative is ensuring the thorough fixation of the whole body for the analysis of multiple organs. However, this fixation method limits tissue availability for other assays, such as western blot or RNA sequencing, which require fresh tissue. By contrast, other methods of euthanasia, such as CO_2_ or isoflurane inhalation followed by subsequent cervical dislocation, allow for the collection of multiple tissues for various analyses from the same animal, making these approaches attractive. To determine whether common anesthetic agents induce hypoxic conditions, which could alter mitochondrial ultrastructure, we first compared mitochondrial morphology in hippocampal brain tissue obtained from wild-type (WT) mice anesthetized with either CO_2_ (3 min) or 5% isoflurane/oxygen prior to cervical dislocation (Figure 4A–C). The whole brain was quickly removed, and hippocampal tissue (3 × 3 × 1 mm) was dissected and immediately immersed in Trump’s solution for fixation (Figure 4A). Consecutive serial tissue sections from hippocampi subjected to each anesthetic technique were examined using TEM. We found that CO_2_ inhalation induced a prominent MOAS phenotype (Figure 4B). Tissue prepared following the inhalation of 5% isoflurane/oxygen did not exhibit significant MOAS formation, containing uniformly elongated mitochondria (Figure 4C).

We next examined how different methods of transcardial perfusion affect mitochondrial morphology in brain tissue (Figure 4D–F). WT mice were anesthetized with ketamine/xylazine injection and perfused with either PBS followed by 4% paraformaldehyde (PFA) or 4% PFA alone without a PBS flush (Figure 4D). Although the PBS/PFA procedure typically requires ≈5 min per mouse, perfusion with PFA alone can be performed in less than 3 min. Intact brains were removed and post-fixed overnight at room temperature in Trump’s solution. The hippocampal CA1 region and cortex was dissected from each brain and processed for TEM. The perfusion of animals with PBS before PFA fixation resulted in MOAS formation in hippocampus and cortex (Figure 4E, hippocampus is shown). By contrast, animals perfused with PFA alone without a prior PBS flush presented uniformly elongated mitochondria throughout the examined brain regions (Figure 4F). These data suggest that transcardial perfusion with a PBS flush before fixation may create hypoxic conditions that affect mitochondrial morphology.

The simple tissue immersion fixation technique is often used instead of a whole-body fixation by cardiac perfusion. The success of immersion fixation depends on the speed at which the fixative penetrates the tissue. If the tissue is too large, a slow fixation process could reduce tissue oxygenation leading to hypoxia, which could alter mitochondrial morphology. To determine the optimum conditions for immersion fixation, we compared two sets of brain tissue obtained from the same WT mouse (Figure 4G). The mouse was euthanized by cervical dislocation without prior anesthesia, and the intact brain was rapidly removed (<2 min). One hemisphere was sliced into 1-mm-thick sections (Figure 4H), whereas the other was cut in half (Figure 4I). All tissues were immediately immersed in McDowell Trump’s fixative solution overnight. After this, the hippocampal CA1 region and cortex were dissected from the 1-mm-thick tissue slices and from the middle of each half of the other hemisphere (Figure 4H,I) and processed for TEM. Mitochondria from the 1-mm-thick slices were ≈0.3 μm in diameter and were uniformly elongated in all brain regions examined (Figure 4H, hippocampus is shown). Mitochondria from the larger brain tissue samples exhibited a wide variety of shapes, including MOAS, in all regions examined (Figure 4I, hippocampus is shown). These data suggest that the immersion fixation of large pieces of tissue may lead to altered mitochondrial morphology. Taken together, our findings suggest that suitable anesthetic agents, such as ketamine/xylazine or 5% isoflurane/oxygen, must be used to reduce artifacts and achieve high quality sample preservation when preparing tissue to study mitochondrial morphology using TEM. We found that CO_2_ should be avoided and if cardiac perfusion must be performed, a PBS flush should also be avoided. For the immersion fixation of fresh tissue, the tissue should not exceed 1 mm in thickness.

### 2.6. 3D EM Methodology

Measurements conducted using 2D TEM micrographs demonstrated alterations in the cristae morphology following *Opa1* and *Mfn2* ablation. However, there are important mitochondrial architectural features that are not visible in 2D and require 3D EM techniques.^[1,11,21–23,25,33,43,67]^ Thus, we utilized FIB- and SBF-SEM to further characterize mitochondrial ultrastructural and morphological changes following gene deletion. These two techniques are similar in allowing for the automated acquisition of serial-section imaging data that can be reconstructed in 3D to provide a detailed, geometrically accurate view of cellular ultrastructure. Both techniques use a similar slice-and-view approach but differ in their fields of view and 3D resolution. While SBF-SEM allows imaging of a large field of view, the precise sectioning capability of FIB-SEM is instrumental for visualization of rare cellular events in a large tissue volume with higher resolution. These two EM platforms could be used independently or in synergy for multiple structural studies.^[46,68]^ Beyond these traditional methods, serial-section procedures for TEM are becoming increasingly automated making these new techniques attractive for research applications. Serial section TEM is slightly different than FIB-SEM and SBF-SEM in offering higher x- and y- resolution allowed for by TEM techniques, which can be necessary in some cases.

**Figure 5.**
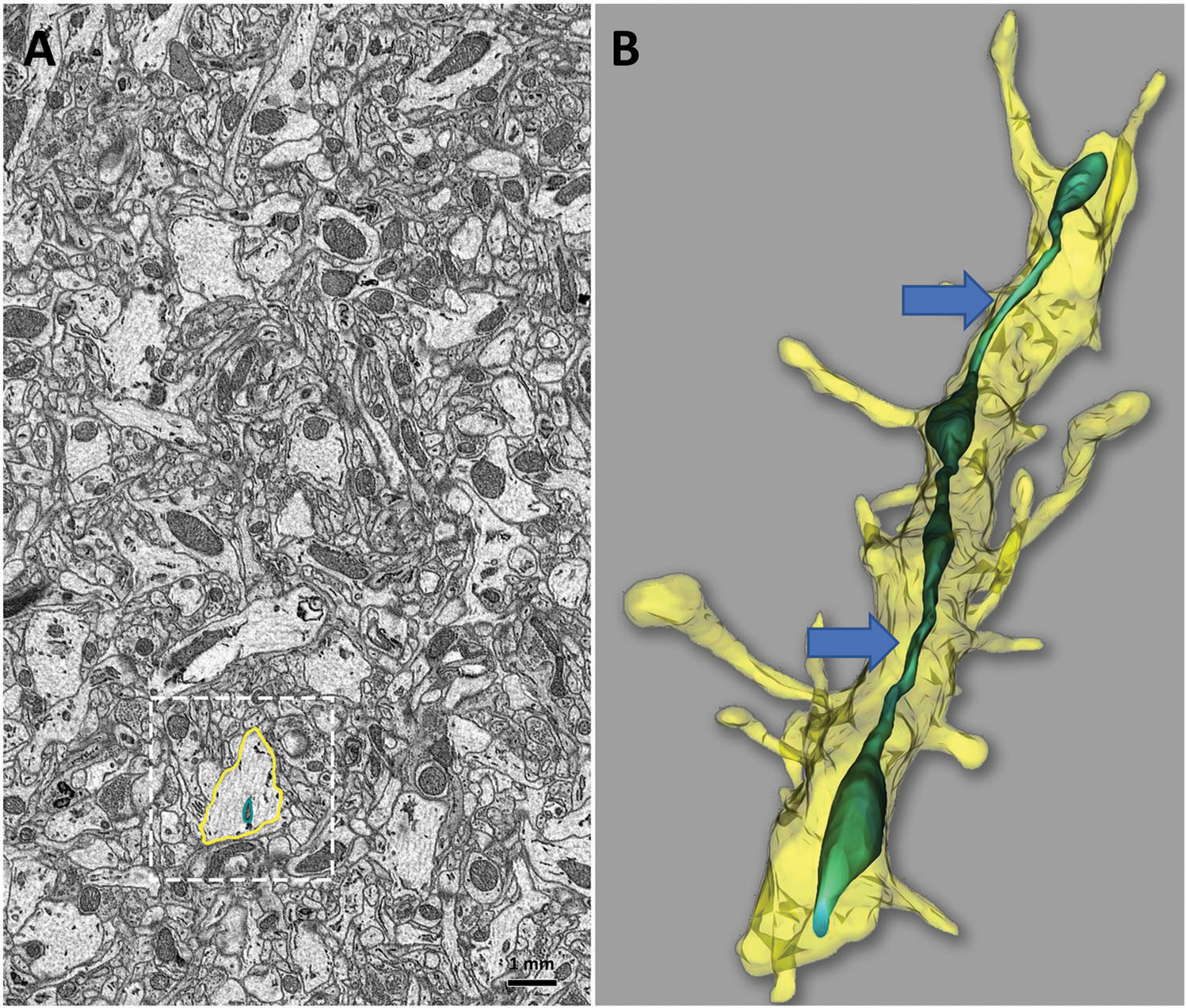
Serial sectioning and 3D EM reconstruction provide additional insight into ultrastructural details of mouse brain tissue that cannot be fully observed in 2D. A) Minimal details of a single neuropil and mitochondria are observed in a 2D micrograph of mouse hippocampal tissue (A, boxed area). B) 3D EM reconstruction of the same brain area using serial block-face imaging allows identifica neuropil as a dendrite, based on the presence of dendritic spines and reveals mitochondrial ultrastructure as MOAS (blue arrows). Reconstruct software was used for 3D reconstruction. Scale bar: 1 mm.

We tested whether techniques developed for the preservation of mitochondrial morphology in brain tissue for 2D EM (Figure 4) are also suitable for 3D SBF-SEM. The APP/PS1 mouse model of AAD), was ev sacrificed by cervical dislocation; hippocampal brain tissue was dissected and 1 mm sections preserved by immersion fixation as described above.^[69]^ Serial-section TEM micrographs were aligned in an image stack (Figure 5A), and the Reconstruct software package was used to visualize the 3D mitochondrial morphology (Figure 5B). As previously demonstrated,^[25]^ MOAS that could not be seen using 2D TEM could be clearly identified using 3D EM in the dendrite of the hippocampal neuron of the APP/PS1 mouse (Figure 5B). Using this technique and frozen brain tissue from the same mice, we were able to combine the EM examination, western blot, and RNA sequencing analyses to reveal the effect of experimental drugs on mitochondrial dynamics and function in AD mouse models.^[69]^ Thus, we provide optimized methods that allow accurate analysis of mitochondria in tissue using 2D or 3D EM without a requirement for a whole-body fixation.

### 2.7. Assessment of MERCs in *Drosophila* and Myotubes Using SBF-SEM

**Figure 6.**
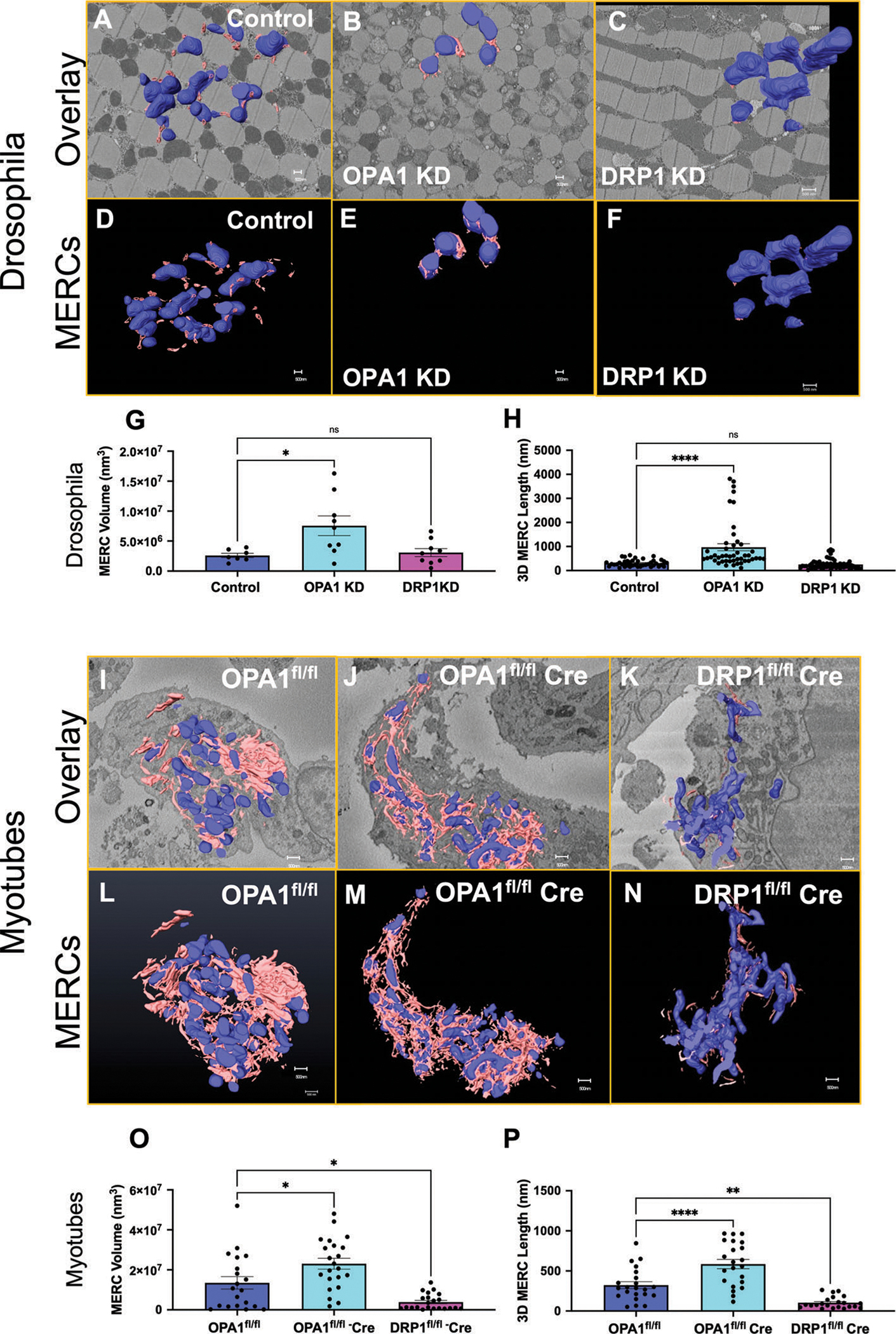
*Opa1*-like-deficiency in skeletal muscle leads to the formation of MERCs in *Drosophila* and mouse myotubes while *Drp1*-like deficiency results in reduced MERCs. 3D reconstruction of mitochondria (blue) and the ER (pink) using SBF-SEM image stacks of A–F) control, *Opa1*-like KD, and *Drp1*-like KD *Drosophila* indirect flight muscle fibers and I–N) primary myotubes. G) MERCs volume and H) length were significantly increased in *Opa1*-like KD but were not affected in *Drp1*-like KD in *Drosophila* skeletal muscles compared to WT. O) MERCs volume and P) length were significantly increased in *Opa1*-like KO primary myotubes but were decreased in *Drp1*-like KO compared to WT. Significance was determined using a Mann–Whitney test. *p < 0.05, **p < 0.01, ***p < 0.001, ****p < 0.0001. SBF-SEM reconstructions from 7 to 23 fully constructed mitochondria, ER, or MERCs. *Opa1*^*fl/fl*^ represents a control while *Opa1*^*fl/fl*^-*cre* represents floxed deletion of *Opa1*.

We next utilized SBF-SEM to determine the effect of the *Opa1*-like or *Drp1*-like knock-down (KD) on MERCs volume, shape, and surface area^[46]^ in *Drosophila* indirect flight muscle in a 3D context (Figure 6A–H and Videos S1 and S2, Supporting Information). The function of the indirect flight muscle is similar to vertebrate cardiac muscle that generates power in an oscillatory manner. The indirect flight muscle is ideal for evaluating the influence of fission/fusion proteins on muscle mitochondria.^[70]^ High-resolution 3D EM reconstructions revealed significant increase in MERCs length and volume in the *Opa1*-like KD muscle relative to the WT (Figure 6A–H and Videos S1 and S2, Supporting Information). Interestingly, the *Drp1*-like-KD fly muscles had no changes in MERCs volume and length relative to control (Figure 6A–H). To explore conservation of these observations, MERCs volume and length were also measured in *Opa1*- and *Drp1*- deficient mouse myotubes (Figure 6I–P). Relative to control myotubes, *Opa1* ablation increased MERCs length and volume while *Drp1* ablation reduced length and volume of MERCs in myotubes (Figure 6I–P and Videos S3 and S4, Supporting Information).Together, these data demonstrate that loss ofOPA1 in mouse primary myotubes and in *Drosophila* muscles increases MERCs volume and length indicating an increase in MERCs tethering. In contrast, the loss of DRP-1 leads to a decrease in MERCs tethering. These data suggest that the optimized fixation protocol is suitable for 3D EM examination of multiple tissues, including mouse and *Drosophila*.

### 2.8. Evaluation of Mitochondrial Size and Morphology in *Opa1*-Like-Deficient *Drosophila* Using SBF-SEM and FIB-SEM

**Figure 7.**
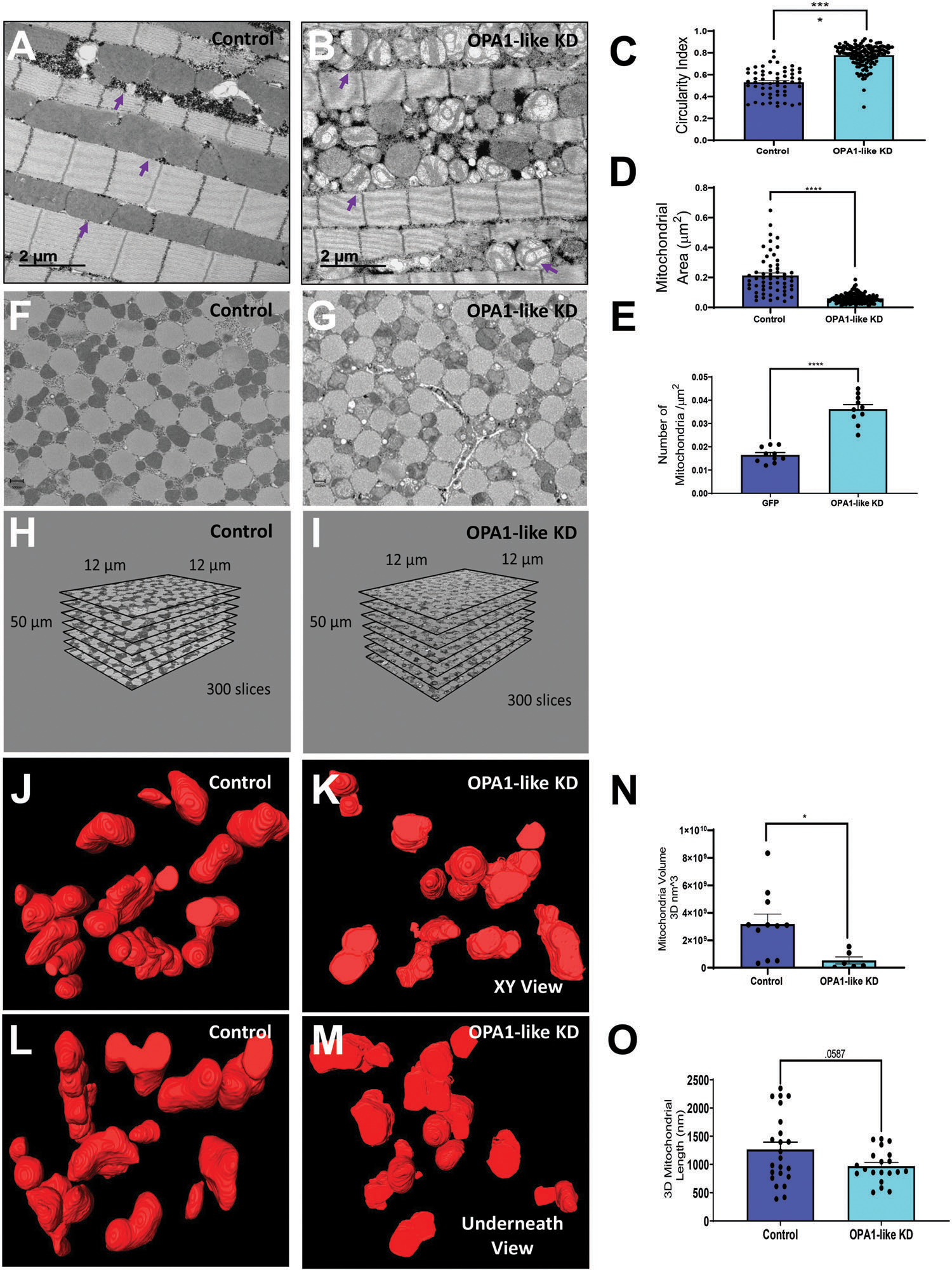
3D EM reconstruction of *Drosophila* indirect flight muscle. A,B) TEM Micrograph of from mitoGFP control (A) and *Opa1*-like KD (B) *Drosophila* flight muscles. Purple arrows identify representative mitochondria. Quantification of C) circularity index, D) mitochondrial area, and E) mitochondrial number in control and *Opa1*-like-KD *Drosophila* flight muscles. Serial block face-scanning EM micrograph of flight muscle mitochondrial morphology from F) control and G) *Opa1*-like KD *Drosophila* flight muscles. Z-stack generated using SBF-SEM from H) control and I) *Opa1*-like KD *Drosophila* muscles used for 3D reconstructions. *XY* 3D reconstruction view of indirect flight muscle mitochondrial morphology from J) control and K) *Opa1*-like KD *Drosophila* using SBF-SEM. Underneath 3D reconstruction view of skeletal muscle mitochondrial morphology from L) control and M) *Opa1*-like KD *Drosophila* using SBF-SEM. Quantification of N) mitochondrial volume and O) mitochondrial length generated using 3D EM reconstruction and SBF-SEM in *Opa1*-like KD *Drosophila* flight muscle compared to control. Significance was determined using a Mann–Whitney test. *p < 0.05, **p < 0.01, ***p < 0.001, ****p < 0.0001.

Previous research demonstrated that the ablation of *Opa1* affected mitochondrial morphology and cristae architecture.^[8,14,71,72]^ We applied our optimized tissue fixation protocol, and used TEM to elucidate the effect of *Opa1*-like ablation on mitochondrial morphology in *Drosophila* indirect flight muscle that expresses mitochondrially targeted green fluorescent protein (mitoGFP) (Figure 7A,B), obtained through previously published methods.^[73]^ We found that *Opa1*-like ablation increased mitochondrial circularity index,^[44,74]^ mitochondrial number, and decreased mitochondrial area (Figure 7C–E). From there, we moved to 3D SBF-SEM reconstruction to allow for viewing of the 3D volume of the mitochondria. Therefore, we also collected data from the skeletal muscle of WT and *Opa1*-like deficient flies (Figure 7F,G) and generated 3D SBF-SEM reconstructions using corresponding Z-stacks (Figure 7H,I). The mitochondrial 3D EM reconstructions viewed from the above or below the *XY* plane are shown in Figure 7J–M. In indirect flight muscle, Opa*1*-like KD resulted in a reduction in mitochondrial volume and length compared to control flies (Figure 7N,O).

**Figure 8.**
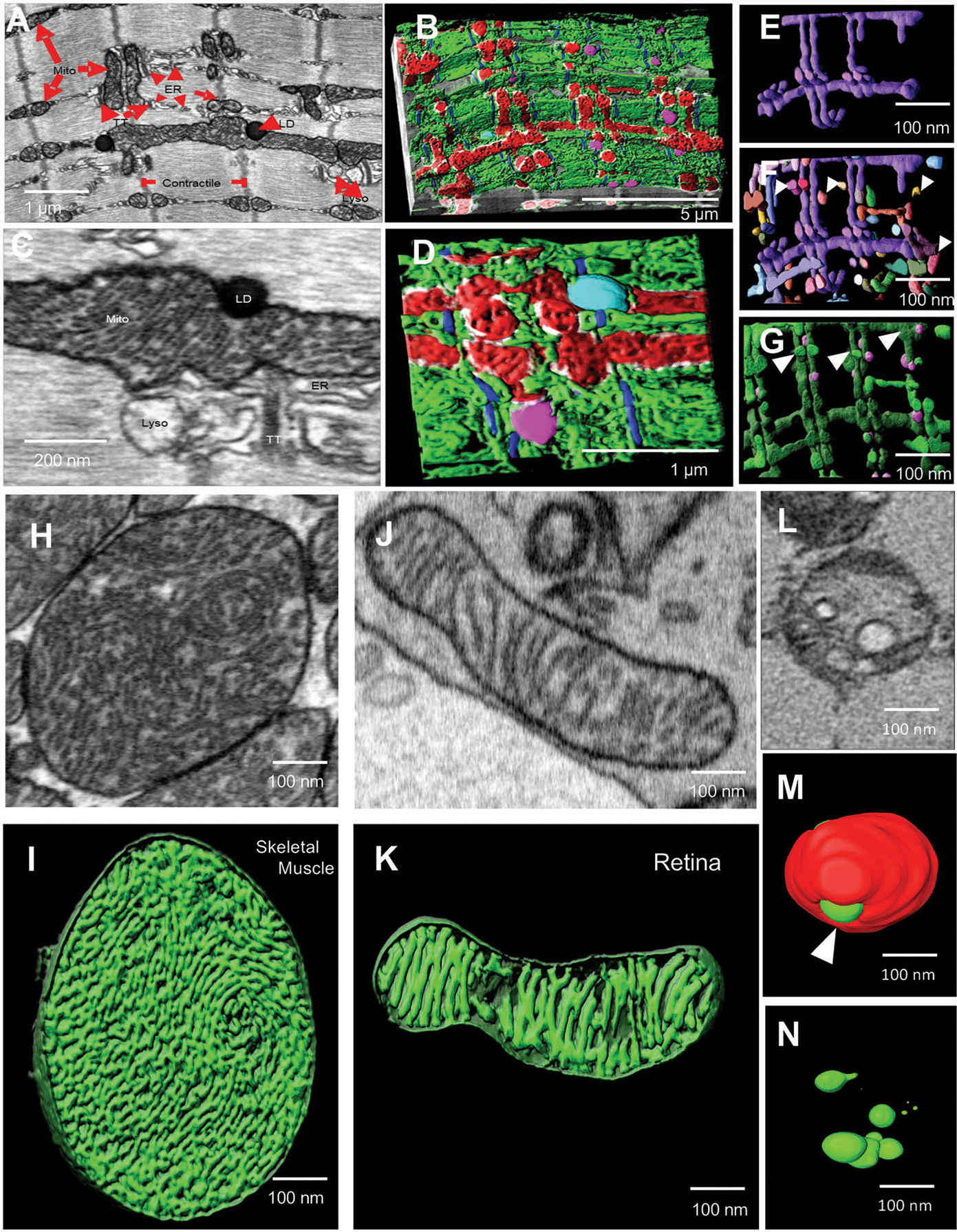
3D reconstruction using FIB-SEM allows identification of complex cellular interactions with high resolution. A–D) TEM micrograph (A) and 3D reconstruction using FIB-SEM (B) of MERCs in mouse skeletal muscle. C) TEM micrograph and D) 3D reconstruction using FIB-SEM of mitochondria (Mito, red), actin (blue), lysosomes (Lyso, pink), lipid droplets (LD, teal), MERCs (white), and ER (green). E–G) 3D reconstruction using FIB-SEM allows the visualization of a single hyperbranched mitochondrion (purple) (E), surrounded by nearby mitochondria (F, various colors, white arrowheads) and lysosomes (G, pink) from adult mouse skeletal muscle. H) TEM micrograph of cristae-containing mitochondria from WT mouse skeletal muscle. I) 3D reconstruction of cristae morphology in WT mouse skeletal muscle using FIB-SEM. J) Micrograph of cristae-containing mitochondria from WT mouse retina tissue. K) 3D reconstruction of cristae morphology in WT mouse retina tissue using FIB-SEM. L) TEM micrograph of a mitochondrion in an *Opa1*-like KD *Drosophila* flight muscle. M) 3D reconstruction of cristae (green) with mitochondria (red) in *Opa1*-like KD *Drosophila* flight muscle. N) 3D reconstruction of cristae morphology (green) in *Opa1*-like KD *Drosophila* flight muscle using SBF-SEM.

Finally, we tested suitability of the immersion fixation protocol for the 3D application using FIB-SEM. As a model, we utilized mouse gastrocnemius muscle. Representative 2D micrographs from the FIB-SEM dataset of a muscle fiber contained clearly identifiable mitochondria, ER, LDs, transverse tubules (t-tubules), and lysosomes (Figure 8A,C). A 3D rendering of the same area allows visualization of the complexity of the interactions of mitochondria with subcellular organelles and structures, MERCs in particular, with high resolution (Figure 8B,D and Video S5, Supporting Information). Application of 3D FIB-SEM enables the generation of unique information visualizing a single hyperbranched mitochondrion (Figure 8E) and its interactions with other mitochondria (Figure 8F) and lysosomes (Figure 8G). Thus, this technique could provide valuable insight into changes in organellar structures and their interactions in tissues in health and disease.

### 2.9. Resolution of Cristae Morphology by FIB-SEM versus SBF-SEM

In cases where mitochondria are tethered by an inter-mitochondrial junction, coordinated changes in mitochondrial cristae morphology between distinct organelles can be observed by TEM.^[44]^ While not all cristae structural details can be observed in 2D micrographs, 3D techniques allow the visualization of fine details. We examined what technique, FIB- or SBF-SEM, provides better resolution to examine fine details of cristae morphology using murine skeletal muscle, retinal tissue, and *Drosophila* flight muscle (Figure 8H–N). Micrographs from a FIB-SEM stack from WT murine skeletal muscle (Figure 8H) and retina (Figure 8J) show crista morphology in representative mitochondria. A 3D rendering of the same tissues allows visualization of cristae morphology with great detail (Figure 8I,K). We next examined the cristae morphology of *Opa1*-like KD *Drosophila* flight muscle using SBF-SEM (Figure 8L–N). A representative micrograph from SBF-SEM stack shows crista resolution (Figure 8L). 3D reconstruction of mitochondrial cristae is shown in Figure 8M,N. Based on limited Z resolution of SBF-SEM, the observation of finer cristae details and morphology, such as lamellar cristae, was better done using FIB-SEM. These data reveal that FIB-SEM can be used to obtain high-resolution details of mitochondrial structure in smaller sample volumes. In contrast, past literature has found that SBF-SEM may be better suited for assessing the relative organellar relationships in large volumes.^[46]^

## 3. Discussion

To realize the full potential of new techniques to assay mitochondrial structure in tissue, sample preparation methods must be reliable and with minimal artifacts. We used serial sectioning TEM reconstruction methods to develop a protocol that could be used to study mitochondrial morphology in various tissue, including brain and muscle. We also demonstrated that procedures that induce hypoxic conditions, including the use of certain anesthetics, perfusion methods, or delayed fixation, are likely to introduce mitochondrial artifacts, such as MOAS. Moreover, our study demonstrated that details of mitochondrial morphology in various tissue could be accurately observed using serial sectioning and 3D EM reconstruction. We developed a comprehensive protocol for designing experiments to promote the most efficient use of animal tissue. It is suitable for the use with both conventional EM techniques based on 2D TEM protocols and advanced techniques such as SBF- and FIB-SEM. In all cases, high-quality micrographs provide rich information on mitochondrial structure and their interaction with other organelles. The complexity of the intracellular environment is not limited to mitochondria. We demonstrated that fixation protocols developed in our study allows preservation of other organelles and cellular structures, including the ER, MERCs, lysosomes, and LDs. The protocol was tested in multiple cells and tissue with high energy demand that rely on mitochondrial function. The outcomes strongly support the suitability of the method to detect changes in mitochondrial morphology, cristae organization, MERCs formation, and subcellular interactions with high accuracy that could be instrumental in multiple research applications. For example, MERCs represent signaling hubs for many molecules, including calcium. Elucidating these interactions may inform the mechanisms underlying diverse pathologies, including neurodegenerative diseases, cancer, diabetes, kidney and liver diseases, and developmental disorders.^[75]^ These technical developments could be important for understanding basic cell biology as cristae are crucial for mitochondrial regulation and function, including oxidative phosphorylation.^[76]^ In addition, these techniques can be applied to determine the best practices for imaging other tissue, such as skeletal muscle in multiple model systems and organisms, such as flies, mice and cultured cells.

While we developed optimized fixation protocols using chemical fixation, we did not consider non-chemical fixation-based techniques that may be relevant to investigating mitochondria dynamics and function. These include high-pressure freezing and freeze substitution, which can quickly stop cell activity to avoid structural artifacts that commonly occur during fixation.^[77]^ This results in a reduction in artifacts compared with traditional EM using chemical fixation.^[78]^ However, high-pressure freezing and freeze substitution remain resource-intensive,^[79]^ making techniques to minimize artifacts in chemical fixation, the most commonly utilized fixation method, still relevant.^[80]^

Along with the development of the optimized fixation protocol, we described multiple methods to assess mitochondrial morphology. Multiple methods exist for analyzing cristae. The cristae score, a scoring system for cristae quantity and form that ranges from 0 (worst) to 4 (best), represents an appropriate measurement for use in tissue. The cristae score is effective as it enables determination whether the cristae structure is intact or has been degraded.^[10]^ However, the cristae score is a subjective judgment that does not incorporate the number of cristae.^[10]^ Thus, the most reliable measurements are cristae volume density, cristae surface area, and cristae number, which are direct measures of changes in the cristae folds or cristae membranes after gene deletion or treatment.^[5,9]^ Mitochondria in tissue can be scored in a similar fashion using mitochondrial volume density and cristae surface area. These parameters can be scored using 2D TEM by examining changes in the typical morphology.^[5,9,10]^ Furthermore, distinguishing which type of cristae was altered by genetic perturbations or treatments is also necessary. Cristae may appear as lamellar or tubular. Tubular cristae present a higher base surface area to volume ratio, whereas lamellar cristae are more capable of expansion and have a higher oxidative phosphorylation potential.^[81,82]^ FIB-SEM allows for 3D reconstruction of the cristae folds, which can be used to distinguish between lamellar and tubular cristae and other diverse mitochondrial phenotypes that may not be observed using 2D EM.^[5,9,10,83]^

The importance of accurate evaluation of MERCs is based on their role in multiple human diseases. *Mfn2* deletion can increase MERCs distance, which was confirmed using our quantification methods. We also used the percentage of the ER and mitochondria coverage to measure the MERCs space.^[19]^ We found that *Mfn2* and *Drp1* ablation increased the ER-mitochondria distance.

Mitochondria adjust to metabolic conditions through the parallel remodeling of cristae and MERCs via OPA1-degrading mechanisms.^[20]^ This occurs in a MFN2-dependent manner, suggesting that MERCs distance can be altered by changes in energy status.^[20]^ Similar to our findings, studies showed that *Mfn2* ablation increased MERCs distance.^[6,17]^ Other studies have reported that the loss of *Mfn2* increases MERCs coverage using various measurements involving the normalization of the MERCs length against either the mitochondrial or the ER surface area.^[19]^ Although other studies have suggested a role of DRP-1 in MERCs,^[55]^ research showing changes in MERCs dynamics after the loss of DRP-1 expression is limited. We present a standardized and systematic method for measuring MERCs and quantifying MERCs distance, and percent coverage, in addition to performing 3D reconstructions. This protocol can be used to further elucidate MERCs dynamics and function. MERCs thickness must be precisely maintained to ensure normal inter-organellar Ca^2+^ transport. Insufficient MERCs distance can result in steric hindrance between the components of the Ca^2+^ transporter machinery. Ca^2+^ uptake between the ER and mitochondria is more likely to occur when the organelles are in close proximity, with an ideal distance ranging from 15 to 30 nm.^[11,12,67]^ For example, smaller MERCs coverage due to larger MERCs distances suggests that adaptations in mitochondria-ER communication in response to metabolic conditions might be negligible.^[1,21]^ However, increased MERCs coverage associated with increased MERCs distance could underlie greater perturbations in the Ca^2+^ uptake or mobilization (the Ca^2+^ transfer effect).^[11]^ Additionally, smaller MERCs distances can allow for the occurrence of lipid transfer.^[11]^ Understanding and accurately quantifying MERCs could therefore inform the understanding of mitochondrial adaptations induced by various diseases and treatments. Similarly, changes in MFN2 or DRP-1 expression can increase ER stress, which induces ER ballooning secondary to increased eukaryotic translation initiation factor 2-alpha kinase and protein kinase R-like ER kinase activity, which are involved in global protein translation attenuation and chaperone expression.^[23]^

## 4. Limitations and Considerations

In this study, we presented fixation and quantification methods that can be used for systematic analysis of organelles in tissues and cells. Unless dealing with a limited data set as was done in this paper, we suggest using a histogram for mitochondrial area. This allows for examination of shifts in the distribution of the mitochondrial area and a better understanding of heterogeneity across mitochondria types.^[5]^ Additionally, a histogram can provide insights regarding changes in mitochondrial size that could occur in response to gene deletion or treatment. Other mitochondrial calculations may not be as viable, which was observed during the 3D reconstruction. For example, when assessing the number of mitochondria per cell or the total mitochondrial volume, we found that the number of mitochondria included in each plane can be a limitation. In general, the total number of mitochondria may be a limiting factor, and 3D reconstruction can limit the number of mitochondria quantified even further due to the long time required for reconstruction and analysis. Therefore, in addition to 2D imaging, we suggest using FIB-SEM more because it may be suitable for determining fine structure of mitochondria or its networks, and SBF-SEM which may provide greater coverage of mitochondria to enable determination of mitochondrial volume density.^[21,28,33,37,41,42,84–86]^ To measure the entire cell, we recommend 3D reconstruction, which provides information on mitochondrial number in the entire cell.

In addition to the imaging techniques described above, other tools can be used to evaluate MERCs tethering, such as the proximity ligation assay (PLA).The PLA permits the detection of protein–protein interactions in situ at distances of less than 40 nm at endogenous protein levels. Co-immunoprecipitation, ER Tracker, and MitoTracker analyses can also be used to examine changes in MERCs colocalization under various experimental conditions.^[17,19,22,43]^ The ER Tracker is an ER-specific dye that shares some overlap with mitochondria. Mitochondrial staining can be used to confirm differences in the ER/mitochondrial colocalization with changes in TEM analyses. MERCs distance can be measured by examining the the ER-mitochondria contact space distance or coverage using FIB-SEM^[1]^ or electron tomography.^[21]^ Immunogold labeling^[22]^ can also be used by individually staining proteins associated with MERCs tethering or mitochondria. The colocalization of these immunogold-labeled dots can also be examined. This technique can be harnessed to validate changes in MERCs tethering protein content,^[21]^ analogous to insights gained from the PLA.^[43,67]^

## 5. Conclusions

The present study demonstrates an optimized approach for preserving specimens and measuring organelle morphology using 2D and 3D EM imaging. We demonstrate that methods that create hypoxic conditions could introduce unintentional artifacts. We describe optimal conditions for fixing cells to preserve cellular and mitochondrial integrity. Furthermore, this study presents standardized quantification methods that can be used to measure mitochondrial morphology. It can further be used to measure other organellar structural features, including mitochondrial size and MERCs. Finally, we verified previously described phenotypes to illustrate efficacy of our methodology. Using these methods, investigators can accurately and reproducibly visualize and measure ultrastructural changes in cells and tissues using TEM imaging techniques.

Beyond MOAS, other mitochondrial morphological phenotypes may arise that require proper resolving and preparation techniques to observe. This is specifically important for mouse and *Drosophila* tissues that were reported to have different mitochondrial shapes.^[83]^

## 6. Experimental Section

### Animals:

All procedures were performed using humane and ethical protocols approved by the Institutional Animal Care and Use Committees from the Mayo Clinic, University of Iowa, and/or National Heart, Lung, and Blood Institute, in accordance with the National Institute of Health’s Guide for the Care and Use of Laboratory Animals. Mice were housed on a 12 h light/dark cycle and were provided access to food and water. C57BL/6 (WT) female mice of various ages were used in the study, with four to six animals examined for each condition. For definitive MOAS identification (positive control), APPSWE (human APP 695 gene containing the double mutations: K670N, M671L) female mice (*n* = 3), a model of familial Alzheimer’s disease, were used.^[87]^ A total of 16 male mice were used to isolate primary satellite cells from *lox-Opa1* mice 8 mice each were used as control littermates (*lox-Opa1* mice) and 8 *Opa1-HSA-CreER*^*T2*^. were used to generate OPA1 deficient myotubes after tamoxifen treatment. All mice were on a pure C57BL/6J genetic background.

### Drosophila Strains and Genetics:

Genetic crosses were performed on a yeast corn medium at 22 °C unless otherwise stated. *Mef2-Gal4* (III) was used to drive the muscle-specific *Opa1*-like (OPA1) (BS #32358) knockdown (KD). *Tub-Gal80ts* (BS #7019) and *Mef2 Gal4* (BS #27390) were used for the conditional muscle-specific *Opa-1*-like KD. Genetic crosses were set up at 18 °C and then shifted to 29 °C at the larval stage (L3). *Mef2 Gal4*; *UAS-mito-GFP* (II chromosome) (BS #8442) was used as control. Stocks were obtained from the Bloomington *Drosophila* stock center. All chromosomes and gene symbols are as described in FlyBase (http://flybase.org).

### Primary Cell Culture:

Satellite cell isolation was performed as previously described.^[47]^ Satellite cells from *Opa1*^*fl/fl*^ mice were plated on BD Matrigel-coated dishes and activated to differentiate into myoblasts in Dulbecco’s modified Eagle medium (DMEM)-F12 containing 20% fetal bovine serum (FBS), 40 ng mL^−1^ basic fibroblast growth factor, 1× non-essential amino acids, 0.14 mm *β*-mercaptoethanol, 1× penicillin/streptomycin, and Fungizone. Myoblasts were maintained with 10 ng mL^−1^ basic fibroblast growth factor and differentiated in DMEM-F12 containing 2% FBS and 1× insulin–transferrin–selenium when 90% confluency was reached. 3 days after differentiation, myotubes were infected with an adenovirus expressing GFP-Cre to achieve *Opa1* deletion. Adenoviruses were obtained from the University of Iowa Viral Vector Core facility. Experiments were performed between 3 and 7 days after infection.

### Measuring Organelle Morphology:

To perform unbiased morphometric analysis of organelles, separate individuals should be responsible for conducting the fixation and image acquisition, while a group of blinded individuals should be responsible for quantification.^[44]^ ImageJ, an open-source image processing software developed by the National Institutes of Health (NIH) and designed to analyze multidimensional scientific images such as TEM and confocal microscopy data sets, was suitable for the quantification analysis of metrics, including length, area, perimeter, and circularity index. Circularity index specifically was a measure of roundness calculated by 4*π* × (*A*/*P*^2^) where *A* was the area and *P* was the perimeter of the desired organelle.^[44,74]^ The entire cell was divided into quadrants, using the ImageJ plugin quadrant picking (https://imagej.nih.gov/ij/plugins/quadrant-picking/index.html), and all measurements should be performed with a minimum of ten cells (*n* = 10) in each of three independent analyses.^[44]^ All cells were selected randomly to avoid bias. For these specific quantification methods, please refer to Lam et al.^[44]^

### TEM Processing of Skeletal Muscle Myoblasts:

To delineate essential calculations for analyzing TEM images, mitochondrial and other organelle morphology in human, primary mouse, and immortalized mouse skeletal myoblasts were investigated. Skeletal muscle satellite cells were isolated, cultured to the myoblast state, and placed in 6-well poly-d-lysine–coated plates for TEM processing. Media was substituted with 2.5% glutaraldehyde in 0.1 m sodium cacodylate buffer (pH 7.2) at 37 °C and incubated for 30 min. Differences in incubation or initial processing are summarized in Figure 1. Subsequent processes were performed directly in the culture plate or directly in fixative at room temperature. After fixation, cells were rinsed twice with 0.1 m sodium cacodylate buffer (pH 7.2) for 5 min each. Next, a secondary fixation was performed using 1% osmium tetroxide + 1.5% potassium ferrocyanide in 0.1 m sodium cacodylate buffer (pH 7.2) for 30 min. The plate was washed repeatedly with 0.1 m sodium cacodylate buffer (pH 7.2) until the liquid appeared colorless. The plate was then washed twice with the same buffer and twice with deionized water (diH_2_O) for 5 min each. Subsequently, the plate was incubated in an en-bloc staining solution of 2.5% uranyl acetate overnight. Dehydration was performed the following day using a graded ethanol series—25%, 50%, 75%, 95%, and two changes of 100% ethanol for 5 min each. Infiltration with an epoxy resin, Eponate 12 (Ted Pella Inc., cat #18005), was performed by gently and thoroughly mixing Eponate 12 with 100% ethanol in a 1:1 solution, replacing the dehydrant with the mixture and incubating for 30 min. The infiltration procedure was repeated three times using 100% Eponate 12 for at least 1 h each. Finally, the intact plate was placed in fresh media in a 70 °C oven for at least 24 h to cure.

After hardening, the plates were removed from the oven, and the resin-embedded cells were separated from the plastic by cracking the plate and immersing the plate in a liquid nitrogen bath. The temperature differential caused by removal from the bath created a gas layer between the cell-embedded resin and the plate, which was then broken. A jeweler’s saw was used to cut an en-face block fitting into the Leica UC6 ultramicrotome (Leica Biosystems) sample holder. The section was then placed onto formvar-coated (Electron Microscopy Sciences, cat #15810) copper grids. The grids were counterstained in uranyl acetate and Reynold’s lead citrate for 2 min each. The samples were imaged using a JEOL 1230 TEM (JEOL Ltd.) at 120 000× with an accelerating voltage. This protocol could also be used to process other cell types, such as hepatocytes, endothelial cells, adipocytes, and cardiomyoblasts.

### TEM Processing of Skeletal Muscle Myotubes:

Differentiation of myoblasts on poly-d-lysine–coated plates alters the normal morphology of skeletal muscle myotubes; therefore, Matrigel coating was employed, allowing even distribution and proper morphology of skeletal muscle myotubes. To obtain skeletal muscle myotubes, skeletal muscle satellite cells were isolated, cultured to the myoblast state, and split on Matrigel-coated plates for myotube formation. The processing of cells grown in a Matrigel-coated plate requires changes to the previously described protocol.

First, 2.5% glutaraldehyde in 0.1 m sodium cacodylate buffer (pH 7.2) was warmed to the temperature of the cell culture media. Cell media was replaced with the warmed fixative and incubated for a minimum of 30 min and a maximum of 1 h, depending on the number of myotubes formed. The subsequent steps were performed directly in the culture plate at room temperature, with gentle agitation on a rotator during incubations to minimize the disturbance of the cell layer. After fixation, the cells were rinsed at least three times with 0.1 m sodium cacodylate buffer (pH 7.2) for 10 min each. Secondary fixation was performed using 1% osmium tetroxide in 0.1 m sodium cacodylate buffer (pH 7.2) with 1.5% potassium ferrocyanide for 45 min. Washing was performed with 0.1 m sodium cacodylate buffer (pH 7.2) until the liquid appeared colorless, followed by two washes with the same buffer and three washes with diH_2_O for 5 min each. En-bloc staining using 2.5% uranyl acetate was performed overnight. Dehydration was performed the following day with a graded series of ethanol at 25%, 50%, 75%, 95%, and two changes of 100% ethanol for 10 min each, while ensuring that the cells did not dry out. Infiltration was performed with an epoxy resin, Eponate 12 (Ted Pella Inc., cat #18005) by gently and thoroughly mixing Eponate 12 with 100% ethanol in a 1:1 solution and replacing the dehydrant with the mixture, allowing infiltration for 1 h. Cells were additionally infiltrated with three incubations in 100% Eponate 12 for at least 2 h for each change of solution. One of the changes was performed overnight at 4 °C. Finally, the intact plate was placed in fresh media in a 70 °C oven for at least 24 h to cure. Extraction from the plates, cutting, staining, and microscopy were performed as described in the preceding section.

### TEM Processing of Mouse Brain Tissue:

Processing of mouse brain tissue for TEM was performed in a BioWave 34700 laboratory microwave oven (Ted Pella, Inc., Redding, CA). Dissected tissue pieces were transferred to microwavable vials following perfusion and initial fixation. All proceeding steps were performed in the BioWave using the parameters outlined below (Table 1):

Following infiltration, tissue was embedded in 100% Embed 812/Araldite resin and allowed to polymerize at 60 °C overnight. Ultrathin sections (90–100 nm) were collected, post-stained with lead citrate, and imaged on a JEOL 1400+ at 80 kV, equipped with a GatanOrius 832 camera.

### Focused Ion Beam-Scanning Electron Microscopy Processing of Adult Skeletal Muscle Fibers:

To reveal muscle cell organelle connectivity in 3D, the morphology and interactions between mitochondria, ER, LDs, lysosomes, and t-tubules in mouse gastrocnemius muscle were analyzed using a protocol that highlights the organelle membranes within the cell. Male C57BL/6J mice were placed on a heated bed and anesthetized via inhalation of 2% isoflurane. Hindlimb hair and skin were removed, and hindlimbs were immersed in 2% glutaraldehyde in 100 mm phosphate buffer, pH 7.2. After 30 min, the gastrocnemius muscle was removed, cut into 1 mm^3^ cubes, and placed in 2.5% glutaraldehyde, 1% PFA, 120 mm sodium cacodylate, and pH 7.2–7.4 for 1 h. After five 3-min washes with 100 mm cacodylate buffer at room temperature, samples were placed in 3% potassium ferrocyanide, 200 mm cacodylate, 4% aqueous osmium for 1 h on ice, washed five times for 3 min each in diH_2_O, and incubated for 20 min in fresh thiocarbohydrazide solution at room temperature. Samples were then incubated on ice for 30 min in 2% osmium solution and washed five times for 3 min each in bi-distilled H_2_O. The sample was then incubated overnight in 1% uranyl acetate solution at 4 °C, washed five times for 3 min each in bi-distilled H_2_O, incubated in 20 mm lead nitrate, 30 mm aspartic acid, pH 5.5 at 60 °C for 20 min, and washed five times for 3 min each in bi-distilled H_2_O at room temperature. The sample was next incubated sequentially in 20%, 50%, 70%, 90%, 95%, and 100% ethanol for 5 min each, incubated in 1:1 Epon:ethanol solution for 4 h, and incubated in 3:1 Epon:ethanol at room temperature overnight. The next day, samples were incubated sequentially in fresh 100% Epon for 1 and 4 h. After removing excess resin using filter paper, the samples were placed on aluminum Zeiss SEM Mounts (Electron Microscopy Sciences, #75510) in a 60 °C oven for 2 days. Stubs were then mounted on a Leica UCT Ul-tramicrotome (Leica Microsystems Inc., USA) and faced with a Trimtool 45 diamond knife (DiATOME, Switzerland), with a feed of 100 nm at a rate of 80 mm s^−1^.

FIB-SEM images were acquired by a Zeiss Crossbeam 540 using Zeiss Atlas 5 software (Carl Zeiss Microscopy GmbH, Jena, Germany) and collected using an in-column energy selective backscatter with filtering grid to reject unwanted secondary electrons and backscatter electrons up to a voltage of 1.5 kV at a working distance of 5.01 mm. Milling was performed by an FIB, operating at 30 kV, with a 2–2.5 nA beam current and 10 nm thickness. Image stacks were aligned using Atlas 5 software (Fibics) and exported as TIFF files for analysis. This protocol, designed for evaluating organelle connectivity in adult skeletal muscle fibers, also works for cardiomyocytes, neonatal myocytes, and brain tissues.^[20]^

### Analysis of FIB-SEM Images for Adult Skeletal Muscle Fibers:

To evaluate 3D organelle connectivity within the FIB-SEM volumes, first, each type of cellular structure were separated within the greyscale datasets. After normalizing contrast throughout the dataset using the 3D Enhance Local Contrast tool in ImageJ (NIH, Bethesda, MD, ImageJ.net) and saving it as an HDF5 (Hierarchal Data Format 5) file, organelle segmentation was completed with the Pixel Classification module in the Ilastik software package^6^ (Ilastik.org). The raw HDF5 file was imported into the software, and the features used to train the pixel classifier were as follows: Color/Intensity 0.3–10 pixels; Edge 0.7–10 pixels; and Texture 0.7–10 pixels. Training of the pixel classifier was performed by tracing the organelle and contractile structures in the middle *XY*, *XZ*, and *YZ* planes of the volume. Training labels were created for the mitochondrial outer membrane, mitochondrial interior, LDs, sarcoplasmic reticulum, t-tubules, lysosome membrane, lysosome interior, and the sarcomeric I-bands, A-bands, and Z-disks. After tracing, the Live Update tool was used to compare the segmentation results to the raw data to check for errors. When needed, further training iterations were performed to reduce errors. Pixel probability maps were then exported as 8-bit HDF5 files.

Segmentation of individual mitochondria and lysosomes was more accurate than initial bulk segmentation; thus, additional training was performed. This second step was performed using the MultiCut module in Ilastik. Raw data was loaded for pixel classification, and then the HDF5 file containing the outer mitochondrial membrane or lysosome membrane probabilities was loaded. Superpixels were created using the membrane probabilities as the input channel, a threshold of 0.2, a presmooth before seeds value of 1.0, clustered seed labeling, and default other values. The training was then performed by marking mitochondrial or lysosomal boundaries in red and non-mitochondrial or lysosomal boundaries in green and selecting the means of the raw data and membrane probabilities standard (edge) and standard (sp) as features. The Live Predict tool was used to create initial boundary predictions before using the Live multicut tool with the Nifty_FmGreedy solver and 0.5 beta to generate the initial individual mitochondrial or lysosomal segmentations. Increased accuracy was obtained by additional boundary training and updating the multicut, when necessary. The multicut segmentation was then exported as an 8-bit HDF5 file for evaluation. Notably, this image segmentation routine works well for cardiomyocytes, neonatal myocytes, myoblasts, satellite cells, brain, retina, and red blood cells, among other tissues.^[20]^ For certain tissues, special instrumentation may be required. For, brain regions, which must be dissected and examined separately, a 1 mm Coronal Mouse Brain Slicer from Abcam (ab243315) was used. 3D renderings of segmented cellular structures were performed using the Volume Viewer plugin in ImageJ.

### Serial Block-Face-Scanning Electron Microscopy Processing of Drosophila Muscle Fibers:

Samples for SBF-SEM were prepared using a procedure based on that developed by Deerinck.^[88]^ Briefly, fresh sample was fixed by immersion in 2% glutaraldehyde + 2% PFA in 0.1 m cacodylate buffer containing 2 mm calcium chloride. After fixation, sample was rinsed in 0.1 m cacodylate buffer and placed into 2% osmium tetroxide + 1.5% potassium ferracyanide in 0.1 m cacodylate, washed with *n*H_2_O, incubated at 50 °C in 1% thiocarbohydrazide, incubated again in 2% osmium tetroxide in *n*H_2_O, rinsed in *n*H_2_O, and placed in 2% uranyl acetate O/N. The next day, sample was rinsed again in *n*H_2_O, incubated with Walton’s lead aspartate, dehydrated through an ethanol series, and embedded in Embed 812 resin. Based on rOTO stains developed by Willingham and Rutherford,^[89]^ this procedure introduced a considerable amount of electron dense heavy metal into the sample to help provide the additional contrast necessary in SBF-SEM. To prepare embedded sample for placement into the SBF-SEM, a ≈1.0 mm^3^ piece was trimmed of any excess resin and mounted to an 8 mm aluminum stub using silver epoxy EpoTek (EMS, Hatfield, PA). The mounted sample was then carefully trimmed into a smaller ≈0.5 mm^3^ tower using a Diatome diamond trimming tool (EMS, Hatfield, PA) and vacuum sputter-coated with gold palladium to help dissipate charge.

Sectioning and imaging of sample was performed using a VolumeScope 2 SEM (Thermo Fisher Scientific, Waltham, MA). Imaging was performed under low vacuum/water vapor conditions with a starting energy of 3.0 keV and beam current of 0.10 nA. Sections of 50 nm thickness were cut allowing for imaging at 10 nm × 10 nm × 50 nm spatial resolution.^[88–90]^

Image analysis, including registration, volume rendering, and visualization were performed using ImageJ,^[3]^ Reconstruct, and Amira (Thermo Fisher) software packages.

### Serial Block-Face-Scanning Electron Microscopy Processing of Retina Tissue:

Retina tissues were isolated via freeze capture.^[91]^ Liquid propane was chilled with dry ice for ≈15 min before being inverted to ensure a liquid jet. In tandem, 20 mL of solvent composed of 97% methanol and 3% acetic acid was also chilled with dry ice. Once mice were euthanized via the methods outlined above, the eyes were rapidly removed, immersed in chilled propane for 1 min, and quickly transferred to the chilled solvent. From there, sections of tissue 4-μm-thick were obtained using a Leica RM2125Rt microtome.

### Statistical Analysis:

GraphPad Prism software (La Jolla, CA, USA) was utilized for all statistical analyses. The presented SBF-SEM and TEM data were the average of three or more independent experiments with consistent results. The mean value was shown with individual data points, and the standard error was indicated by bars. An unpaired *t*-test was used when comparing two groups (or the non-parametric equivalent of a Mann–Whitney test, if applicable), while one-way ANOVA with Fisher’s protected least significant difference test was used for more than two groups. A significance level of *p* < 0.05 was considered significant (*), while higher levels of significance (*p* < 0.01, *p* < 0.001, and *p* < 0.0001) were displayed as (**, ***, and ****), respectively.

## Supplementary Material

Supplementary Material

Video 5

Video 3

Video 2

Video 1

Video 4

## Figures and Tables

**Table 1.** Processing Steps Performed in BioWave Laboratory Microwave.

Step	Microwave Time (s)	Wattage (W)	Vacuum	Reagent

Wash (3x)	40	250	no	0.1 M phosphate buffer, pH 7.0
1% aqueous osmium	40 on-off-on, 15 min rest	250	yes	
Wash (3x)	40	250	no	0.1 M phosphate buffer, pH 7.0
2% aqueous UA	40 on-off-on, 15 min rest	250	yes	
Wash (3x)	40	250	no	0.1 M phosphate buffer, pH 7.0
Dehydration	40	250	no	60%–100% EtOH
Dehydration	40	250	no	100% HPLC grade Acetone
Infiltration	40 on-off-on, 15 min rest	250	yes	1:2, 1:1, 3:1, Embed 812/Araldite: acetone

## Data Availability

The data that support the findings of this study are available from the corresponding author upon reasonable request.
