# Supplementary material for: A Comprehensive Approach to Sample Preparation for Electron Microscopy and the Assessment of Mitochondrial Morphology in Tissue and Cultured Cells
Source: Adv Biol (Weinh). Author manuscript; Available in PMC 2024 Apr 1. (PMC10615857; doi:10.1002/adbi.202200202)
Supplement: Supplementary Material [file NIHMS1917700-supplement-Supplementary_Material.pdf]

ADVANCED BIOLOGY

Supporting Information

for *Adv. Biology*, DOI 10.1002/adbi.202200202

A Comprehensive Approach to Sample Preparation for Electron Microscopy and the Assessment of Mitochondrial Morphology in Tissue and Cultured Cells

Antentor Hinton Jr., Prasanna Katti, Trace A. Christensen, Margaret Mungai, Jianqiang Shao, Liang Zhang, Sergey Trushin, Ahmad Alghanem, Adam Jaspersen, Rachel E. Geroux, Kit Neikirk, Michelle Biete, Edgar Garza Lopez, Bryanna Shao, Zer Vue, Larry Vang, Heather K. Beasley, Andrea G. Marshall, Dominique Stephens, Steven Damo, Jessica Ponce, Christopher K. E. Bleck, Innes Hicsasmaz, Sandra A. Murray, Ranthony A. C. Edmonds, Andres Dajles, Young Do Koo, Serif Bacevac, Jeffrey L. Salisbury, Renata O. Pereira, Brian Glancy*, Eugenia Trushina* and E. Dale Abel**

Supporting Information

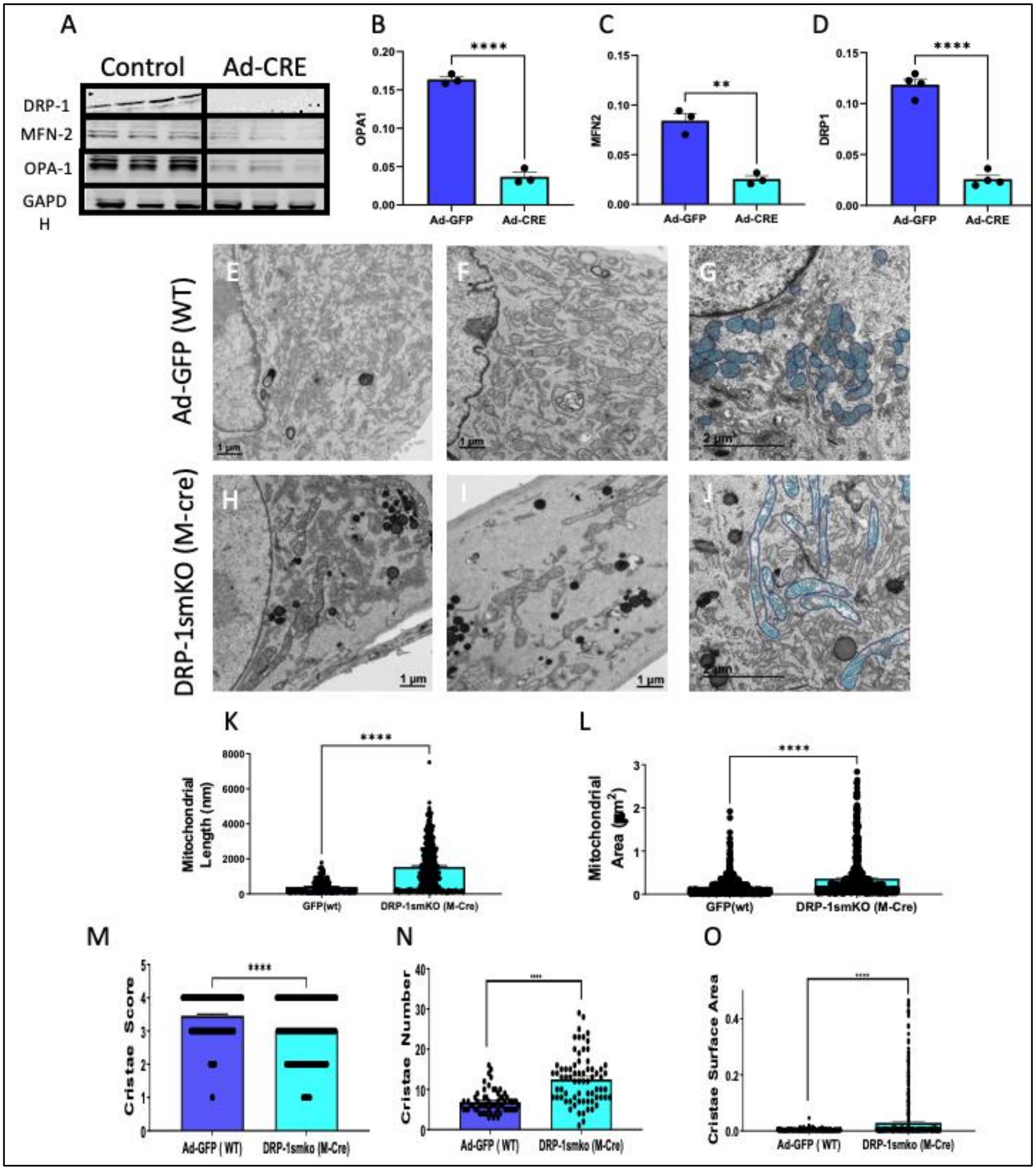

Figure S1. Quantification of mitochondrial characteristics in *Opa1*-deficient skeletal muscle myoblasts and *Mfn2*-deficient or *Opa1*-deficient skeletal muscle myotubes.

A. Western blot shows successful knockout and reduced expression of DRP-1, MFN2, and OPA1. GAPDH was used as a control. **B-D.** Qualitative polymerase chain reaction showed reduced *Opa-1**,** Drp-1* and

Mfn2 gene expression. Ultrastructure of control (**E-G)** and *Drp-1*-deficient (**H-J)** primary skeletal muscle myotubes. Quantification of mitochondria length (**K)**, mitochondria area (**L)**, cristae score (**M)**, cristae number (**N)**, and cristae surface area (**O)** in control and *Drp-1*-deficient primary skeletal muscle myotubes. Significance was determined using a Mann-Whitney *t*-test. ***P* < 0.01, *****P*<0.0001.

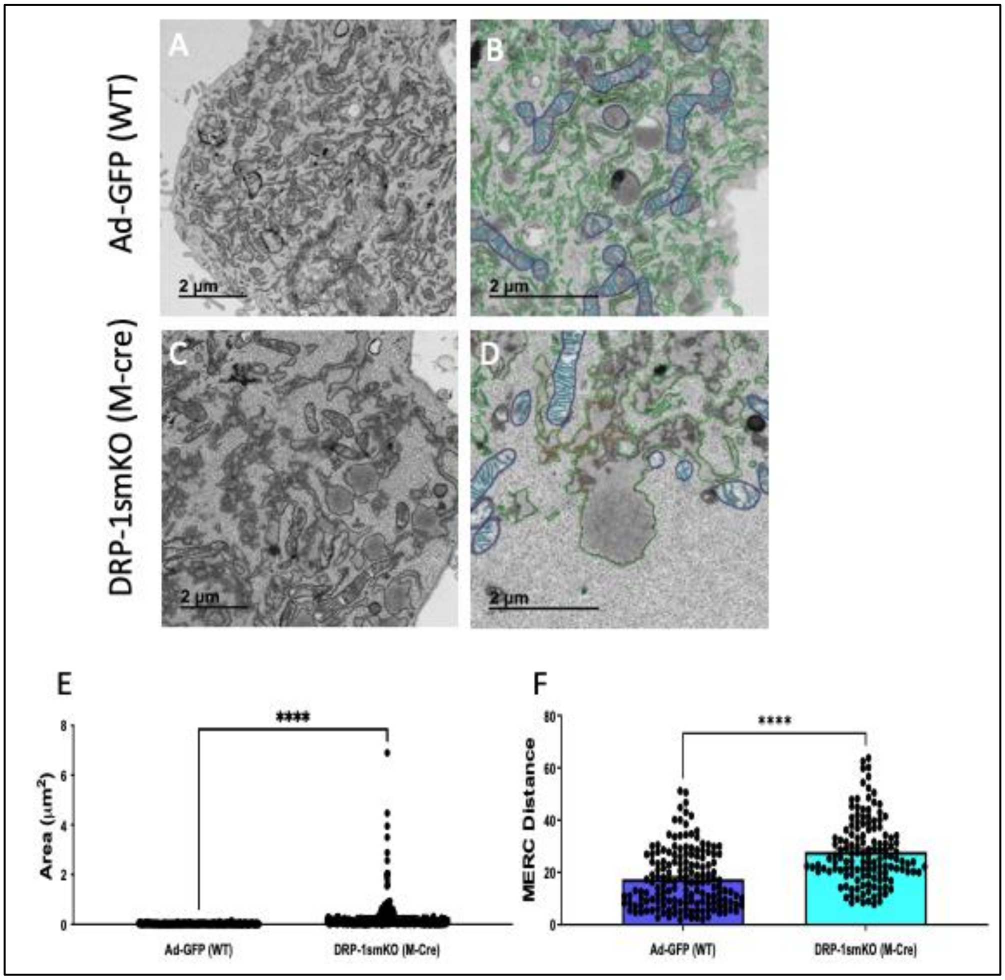

Figure S2. MERCs ultrastructure in *Drp-1*-deficient primary skeletal muscle myotubes.

Ultrastructure of control (**A-B**) and *Drp-1*-deficient (**C-D)** primary skeletal muscle myotubes. Significant increase in area (**E**) and MERCs distance (**F**) were observed in *Drp-1* KO cells compared to control. Significance was determined using a Mann-Whitney *t*-test. **P* < 0.05, ***P* < 0.01, ****P* < 0.001, *****P*<0.0001.

Video S1. 3D reconstruction of MERCs in WT drosophila flight muscle.

Video S2. 3D reconstruction of MERCs in drosophila flight muscle with *Opa1* knockdown.

Video 3S. 3D reconstruction of MERCs in *Opa1*$^{fl/fl}$ mouse myotubes.

Video S4. 3D reconstruction of MERCs in *Opa1*$^{fl/fl}$*-Cre* myotubes.

Video S5. 3D reconstruction of mitochondria membranes (red), ER (green), actin (blue), LDs (cyan), lysosomes (magenta), and MERCs (white space) in WT mouse gastrocnemius muscle.
